# LOX-catalyzed collagen stabilization is a proximal cause for intrinsic resistance to chemotherapy

**DOI:** 10.1038/s41388-018-0320-2

**Published:** 2018-05-21

**Authors:** Leonie Rossow, Simona Veitl, Sandra Vorlová, Jacqueline K. Wax, Anja E. Kuhn, Verena Maltzahn, Berin Upcin, Franziska Karl, Helene Hoffmann, Sabine Gätzner, Matthias Kallius, Rajender Nandigama, Daniela Scheld, Ster Irmak, Sabine Herterich, Alma Zernecke, Süleyman Ergün, Erik Henke

**Affiliations:** 1 Institute of Anatomy and Cell Biology II, Universität Würzburg, Koellikerstrasse 6, 97070 Würzburg, Germany; 2 Institute of Experimental Biomedicine, Universitätsklinikum Würzburg, Josef-Schneider-Strasse 2, 97082 Würzburg, Germany; 3 School of Health Sciences, Bilgi University, 34440 Beyoğlu İstanbul, Turkey; 4 Institute of Tissue Engineering, Universität Würzburg, Roentgenring 11, 97070 Würzburg, Germany; 5 Graduate School of Life Science, Josef-Schneider-Strasse 2, 97082 Würzburg, Germany; 6 Zentrallabor, Universitätsklinikum Würzburg, Josef-Schneider-Strasse 2, 97082 Würzburg, Germany

## Abstract

The potential of altering the tumor ECM to improve drug response remains fairly unexplored. To identify targets for modification of the ECM aiming to improve drug response and overcome resistance, we analyzed expression data sets from pre-treatment patient cohorts. Cross-evaluation identified a subset of chemoresistant tumors characterized by increased expression of collagens and collagen-stabilizing enzymes. We demonstrate that strong collagen expression and stabilization sets off a vicious circle of self-propagating hypoxia, malignant signaling, and aberrant angiogenesis that can be broken by an appropriate auxiliary intervention: Interfering with collagen stabilization by inhibition of lysyl oxidases significantly enhanced response to chemotherapy in various tumor models, even in metastatic disease. Inhibition of collagen stabilization by itself can reduce or enhance tumor growth depending on the tumor type. The mechanistical basis for this behavior is the dependence of the individual tumor on nutritional supply on one hand and on high tissue stiffness for FAK signaling on the other.

## Introduction

The tumor microenvironment comprises the extracellular matrix (ECM), the vasculature, and various tumor-associated immune and stromal cells (TACs). It varies strongly in composition, density, and function from the microenvironment in normal tissue [1, 2]. While these alterations generally point in a certain direction, e.g., toward a more abundant and rigid ECM, or an increasingly dysfunctional vasculature, the microenvironment differs considerably between various tumors [3]. This has important clinical implications as the microenvironment strongly affects the course of malignant diseases and their treatability. The malignantly altered tumor microenvironment is i.a. responsible for the reduced and heterogenic supply that characterizes solid tumors: Intratumoral signaling is strongly shifted toward pro-angiogenic factors, leaving tumor blood vessels in a constant state of re-arrangement and immaturity [4, 5]. Consequently, the vasculature in tumors is often defective and dysfunctional, reducing tumor perfusion [6, 7]. This not only increases malignant behavior, as both hypoxia and metabolic stress enhance invasiveness and metastasis [8, 9], but the poorly supplied areas are also largely protected from therapeutically effective drug concentrations [10–13]. In addition, stromal and immune cells provide growth signals, influence invasiveness, and confer chemoprotection [14, 15]. Thus, modulating the microenvironment bears significant potential as a strategic approach to improve response to therapy.

While the ECM is probably the least studied component of the microenvironment with respect to chemoprotection, several studies have already pinpointed specific protective interactions between the ECM and therapeutic agents. For example, it has been shown that the high water content of hyaluronan-rich pancrease tumors creates a high interstitial pressure, interfering with drug distribution [16, 17]. Likewise, high fibrillar collagen deposition might lead to vascular collapse subsequently obstructing drug supply [18]. Finally, the distribution of therapeutic antibodies is strongly restricted by the ECM [2, 19]. Most of the previous studies focused on either the interaction of ECM components with specific, often targeted drugs, or on particular microenvironmental conditions observed in only a subset of solid tumors. However, it is to be expected that the ECM has a strong, general and rather indiscriminatory effect on the interstitial transport of drugs and thereby on their efficacy.

We used a multi-data set cross-evaluation approach to identify ECM components and modifiers that were correlated with resistance to a wide range of standard non-targeted chemotherapeutic drugs in cancer patients. In a second step, we extracted druggable targets from the obtained results with the aim to evaluate the potential for response-improving auxiliary treatment. This screen identified lysyl oxidases as targetable enzymes critically associated with a subset of resistant tumors characterized by high collagen expression. These results were evaluated in an array of murine tumor models by pharmacological inhibition and ectopic overexpression of lysyl oxidases. In these systems we were able to demonstrate that indeed lysyl oxidases contribute strongly to drug resistance by stabilizing matrix collagen. We demonstrate that the ECM plays an important role in intrinsic drug resistance and that ECM modification is a suitable way to improve drug sensitivity and reduce malignancy.

## Results

### A subset of therapy-resistant tumors is defined by high collagen expression

**Fig. 1:**
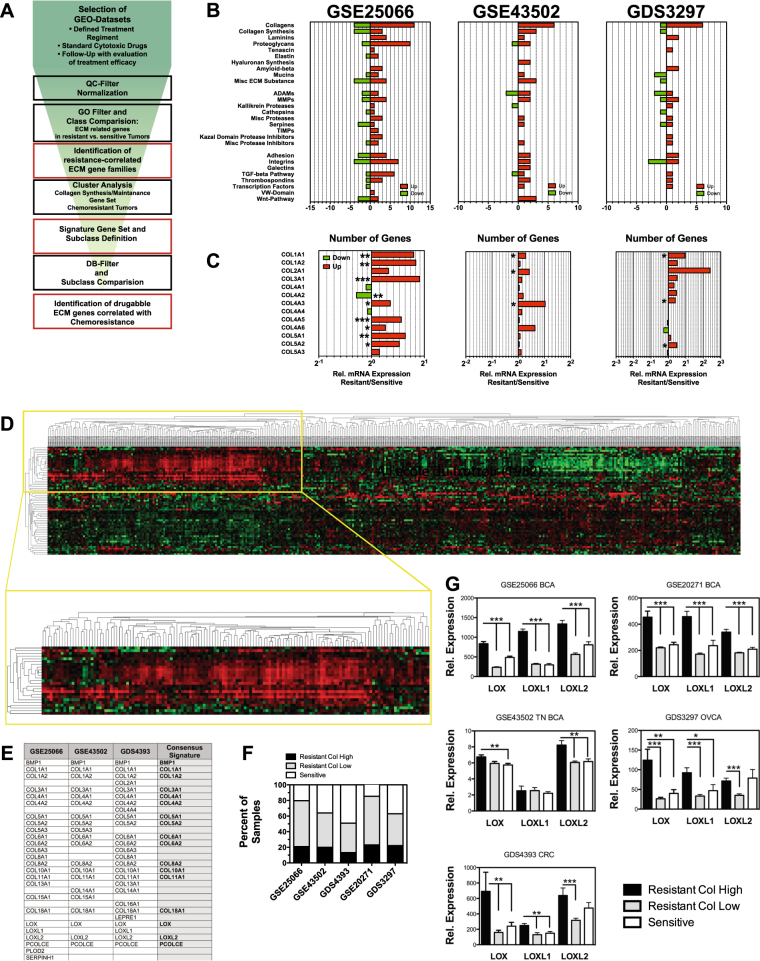
High collagen expression defines a subset of chemoresistant tumors. **a** Schematic representation of workflow in microarray analysis. **b** Number of ECM-related genes with statistically significant (*p* < 0.05) different expression levels in resistant vs. sensitive tumors found in patient data sets. Genes are grouped into the presented families. Absolute numbers of family members with increased (red bars) and reduced (green bars) expression are displayed. **c** Relative expression levels of genes encoding collagens I–V in patient data sets. **d** Cluster analysis of data set GSE25066 for collagen and collagen synthesis genes in samples from resistant tumors. **e** Genes defining cluster with elevated collagen expression in data sets GSE25066, GSE43502, and GSE4393 and derived 17-gene consensus signature. **f** Distribution of patient samples with Res_High_, or Res_Low__-_ signature vs. sensitive tumors in the data sets. **g** Relative expression levels of LOX, LOXL1, and LOXL2 in Res_High_, Res_Low_ and sensitive tumors of patient data sets. Error bars: ±SEM. * indicates statistical significance vs. control, *P < 0.05, **P < 0.01. ***P < 0.001

To identify ECM-related proteins that are linked to therapy resistance, we analyzed microarray data sets of pre-treatment biopsies obtained from ovarian, colon, and breast carcinoma patient groups with available follow-up information on response to subsequent chemotherapy (Supplementary Table 1). Data sets derived from patient cohorts receiving different chemotherapeutic regiments were selected to control for expression differences caused by treatment-specific resistance mechanisms. Using functionally annotated gene sets derived from Gene Ontology (geneontology.org), the data sets were tested for differential expression of ECM-related genes between the classes of sensitive and resistant tumors (Fig. 1a). Chemoresistant tumors appeared to have higher expression of ECM-related genes (Supplementary Tables 2–4). Several ECM-related gene families, including collagens, laminins, and proteoglycans, showed a consistent tendency for higher expression in resistant tumors across the tested data sets (Fig. 1b). Synthesis, maturation, and proteostasis of collagens are well studied and various potentially druggable enzymes that are involved in the build-up and maintenance of the collagen matrix are identified. Thus, focusing on genes of the collagen synthesis pathway had the potential to lead to the identification of effective and utilizable targets. While 25 different collagens are expressed in humans, relative abundance varies strongly. The fibrillar collagens I, II, III, and V along with the major component of the basal lamina collagen IV represent up to 95% of the collagen found in tissue, while other collagens contribute much less to the volume of the ECM. Collagens I–V were significantly upregulated in the resistant tumors of the data sets (Fig. 1c). It is reasonable that only in a subset of resistant tumors resilience to therapeutics is conferred by increased collagen deposition, while in other tumors resistance might be caused by other mechanisms. To identify subsets of tumors with resistance correlated to collagen content, a set of 64 genes comprising all collagens and genes involved in collagen synthesis and modification was defined (Supplementary Table 5) [20]. This gene set was used to stratify the data sets by cluster analysis on arrays of resistant tumors only, leading to the identification of a subset of tumors with significantly increased expression of a cluster of collagens and genes involved in collagen synthesis (Fig. 1d). Three data sets (GSE25066, GSE43502, and GDS4393) were used as training data sets, two (GSE20271 and GDS3721) for later validation of obtained results. Correlation of the analysis of the three training data sets led to the designation of a 17-gene signature (Fig. 1e). This signature was found also within the chemoresistant tumors of the other data sets, defining a group of 26–35% of the resistant tumors (Fig. 1f), while being absent in chemotherapy-sensitive tumors. Consequently, the data sets were subdivided into three subcategories of chemosensitive (Sens), resistant with high expression of the collagen signature genes (Res_High_) and resistant with low collagen signature profile (Res_Low_) tumors. Expression analysis of genes of the collagen synthesis pathway that were significantly elevated in the Res_High_ compared to both the Res_Low_ and to Sens tumors was correlated with the results from corresponding DrugBank (http://www.drugbank.ca) entries to identify druggable targets (Supplementary Table 6). The lysyl oxidases, LOX, LOXL1, and LOXL2, were the most prominent and consistently upregulated druggable genes in the Res_High_ group (Fig. 1g).

### LOX/LOXL2 overexpression reduces growth of 4T1 tumors by limiting their supply

**Fig. 2:**
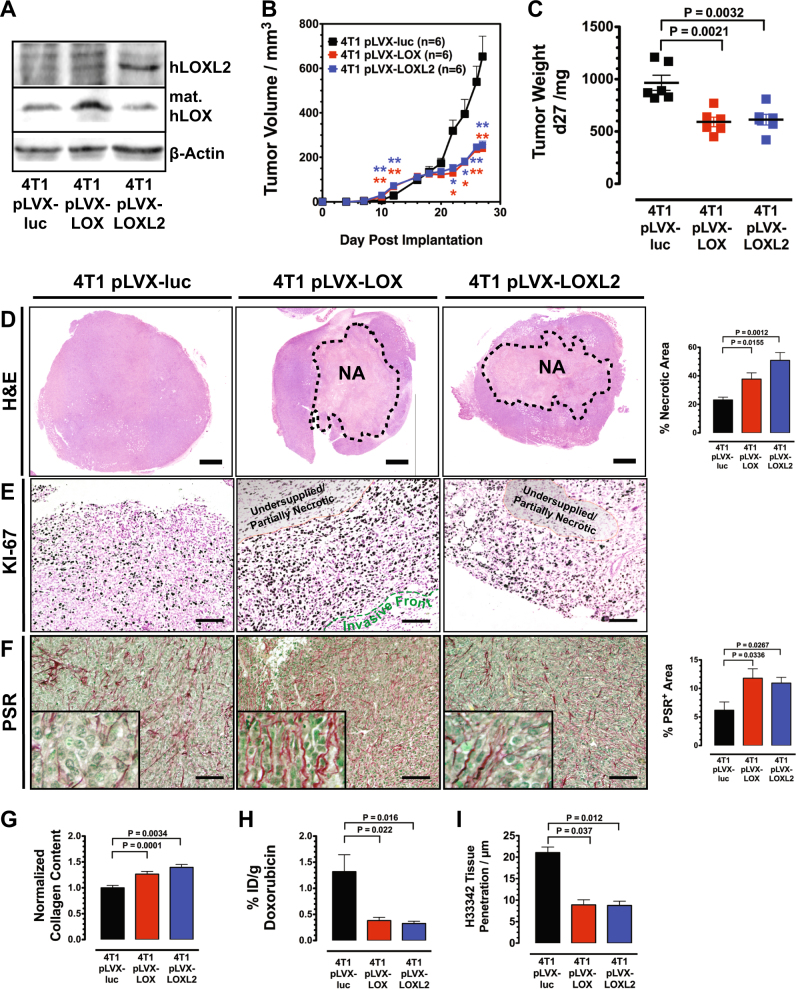
LOX/LOXL2 overexpression reduces supply of 4T1 tumors. **a** Western blot analysis of LOX and LOXL2 expression levels after stable transduction with lentiviral particles pLVX-luc-LOX, pLVX-luc-LOXL2, and the control particles pLVX-luc. **b** Growth curve of breast carcinomas (BCAs) generated by implantation of 4T1-pLVX-luc, 4T1-pLVX-luc-LOX, and 4T1-pLVX-luc-LOXL2 cells. **c** Weight of 4T1 control and 4T1 LOX/LOX2 OE BCAs 27 days after implantation. **d** Histological analysis of H&E-stained 4T1 and 4T1 LOX/LOX2 OE tumors reveals increased necrosis in the central region of 4T1 BCAs after LOX/LOXL2 OE. NA necrotic area, SB: 1000 µm. **e** Ki-67 staining at the front of 4T1 control and 4T1 LOX/LOX2 OE BCAs. SB: 100 µm. **f** Analysis of PSR-stained 4T1 control and 4T1 LOX/LOX2 OE BCAs shows increased staining for fibrillar collagens in 4T1 tumors after LOX/LOXL2 OE. SB: 100 µm. **g** Photometric quantification of Sirius Red bound to collagen in tissue sections. Values are normalized against total protein content. (*n* = 6). **h** Quantification of doxorubicin accumulated in 4T1 control and 4T1 LOX/LOX2 OE BCAs 2 h post injection (*n* = 9). **i** Quantification of H33342 tissue penetration in 4T1 control and 4T1 LOX/LOX2 OE BCAs. Distance of detectable H33342 staining from vessel surfaces in 3D confocal micrographs was measured (*n* = 4). Error bars: ±SEM. * indicates statistical significance vs. control, *P < 0.05, **P < 0.01

Recently it has been shown that highly crosslinked collagen also forms a physical diffusion barrier for small molecules and that it effectively protects tumor cells in 3D cultures from drugs [21]. These results indicate that increased levels of lysyl oxidases that were found in a subset of resistant tumors from the patient data sets, obstruct extracellular diffusion thereby reducing supply of tumor cells with nutrients and oxygen and protecting them from exposure to therapeutic agents. We generated murine 4T1 breast cancer cells that stably overexpressed LOX and LOXL2, respectively, using a lentiviral delivery system to test whether lysyl oxidases indeed conferred a chemoprotective modification of the tumor ECM (Fig. 2a). T1 cells were chosen because they express low levels of the two enzymes, but significant amounts of collagens [21]. Interestingly, while growing initially faster tumors generated from 4T1 cells overexpressing LOX or LOXL2 eventually fell back in their growth rate behind control tumors (Fig. 2b, c). Histological analysis of the implanted tumors after 27 days of growth showed that both LOX and LOXL2 overexpression (OE) led to a massive increase of central necrosis (Fig. 2d). On the other hand, a high abundance of cells positive for the proliferation maker KI-67 was observed in a narrow proliferative rim of ~250 µm at the perimeter or invasive front of the LOX/LOXL2 OE tumors (Fig. 2e). Proliferation decreases sharply toward the undersupplied inner region of the tumors, where partial necrosis was already observed. The OE tumors displayed a denser highly organized network of fibrillar collagen, which comprised individual filaments encapsulated in small clusters of tumor cells (Fig. 2f). LOX/LOXL2 OE not only changed the appearance and characteristics of the collagen fibrils, but also led to increase of overall collagen contend in the tumors (Fig. 2g).

To validate if the observed steep gradient supplied within the LOX/LOXL2 OE tumors also affects drug delivery, we measured accumulation of DOX in the tumors (Fig. 2h). DOX indeed accumulated at significantly reduced amounts in LOX/LOXL2 OE tumors. To test for changes in actual supply with blood-borne molecules, we used Hoechst 33342 (H33342) as a tracer substance. Mice were injected with H33342 and fluorophor-labeled isolectin for vessel visualization prior to killing. Distribution of H33342 in relationship to supplying blood vessels was evaluated by 3D-CLSM. These tumor permeation studies demonstrated that in the LOX/LOXL2 OE tumors only few cell layers surrounding the blood vessels are well supplied with H33342 and that small molecules barely permeate into the dense surrounding tissue (Fig. 2i). Concomitantly, the experiments also showed an increase of perfused vessel volume in the LOX/LOXL2 OE tumors (Supplementary Figure S1)

### Lysyl oxidase inhibition improves tumor supply

**Fig. 3:**
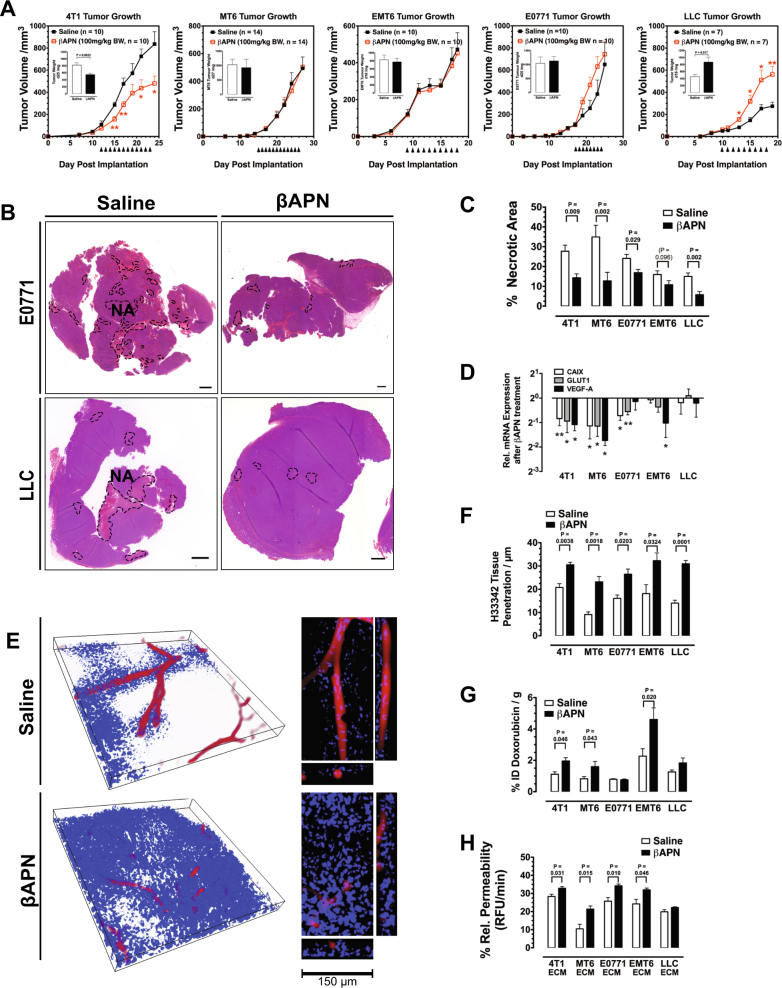
Tumor supply is improved after lysyl oxidase inhibition. **a** Table of tumor models used for evaluation of prolonged lysyl oxidase inhibition and treatment scheme. Fully established tumors (>50 mm^3^) were treated by daily i.p. injections with 100 mg/kg BW βAPN until tumors reached a size of 1000 mm^3^. **b** H&E staining of whole tumor sections in βAPN-treated and control E0771 and LLC tumors. Necrotic areas (NA) are indicated (SB = 1000 µm). **c** Quantitative evaluation of necrotic areas in βAPN-treated and control tumors (*n* = 6–10). **d** mRNA expression analysis of Hif1α target genes *ca-ix*, *vegf-a*, and *glut1* after βAPN treatment using the GeXP system (*n* = 4). **e** H33342 penetration in 4T1 tumor sections after βAPN treatment, 3D rendering, and orthogonal views of selected vessels. Isolectin GS B4-Alexa 647 (red) and H33342 (blue). Size of bounding box: 500 µm × 500 µm × 45 µm. **f** Quantification of H33342 tissue penetration. Distance of detectable H33342 staining from vessel surfaces in 3D confocal micrographs was measured (*n* = 4). **g** Quantification of DOX accumulated in βAPN-treated and control tumors 2 h post injection (*n* = 6–10). **h** Fluorometric quantification of DOX diffusion through ECM isolates from βAPN-treated and control tumors (*n* = 4). Error bars: ±SEM. * indicates statistical significance vs. control, *P < 0.05, **P < 0.01

To evaluate the potential of lysyl oxidase inhibition to increase tumor supply and efficacy of drug delivery, we examined the effect of prolonged lysyl oxidase inhibition on several indicators for hypoxia, metabolic stress, and drug distribution in a range of tumor models. Three-aminopropionitrile (βAPN), an inhibitor of all five lysyl oxidase family members, was used to treat established tumors in five different syngeneic models, 4T1, EMT6 and E0771 breast carcinomas, Lewis lung carcinomas (LLC), and MT6 fibrosarcomas. Treatment of established tumors with βAPN (100 mg/kg/BW ip qd) reduced significant collagen cross-linking in the tumor ECM (Supplementary Figure S2). In contrast to previous studies that reported a solid reduction of growth in various tumor models [22–25], treatment with βAPN reduced tumor growth only in the 4T1 model, while three models (MT6, EMT6, and E0771) did not respond with a change in growth rate and growth of LLC tumors was even strongly increased (Fig. 3a). Despite the different response with respect to growth rate, lysyl oxidase inhibition led to an improved overall supply in all models: tumor sections after βAPN treatment showed reduction of central necrosis (statistically significant in four of the five models), indicating a reduction in hypoxic and metabolic stress (Fig. 3b, c). mRNA expression of molecular markers for hypoxia (*Vegf-a*, *Ca-ix*, and *Glut1*) was also reduced in all but the inversely responsive LLC model (Fig. 3d). In βAPN-treated tumors, H33342 was able to penetrate much deeper into the tissue than in control tumors (Fig. 3e), resulting in a larger proportion of the tumors supplied with the tracer dye (Fig. 3f). The improved supply was also confirmed by an increased accumulation of doxorubicin (DOX) in the treated tumors (Fig. 3g). Perfused vessel volume was reduced in 4T1, MT6, and EMT6 tumors after βAPN treatment showing the opposite effect of LOX/LOXL2 OE (Supplementary Fig. 1). In LLC and E0771 tumors, LOX(L) inhibition did not affect perfused vessel density. To verify that changes in the ECM were causal for the reduced supply, we isolated ECM from treated and control tumors and measured the diffusion rate of DOX through a layer of the isolated ECM deposited on transwell membranes (Fig. 3h). ECM isolates from all tumor models showed improved permeability for DOX after lysyl oxidase inhibition.

**Fig. 4:**
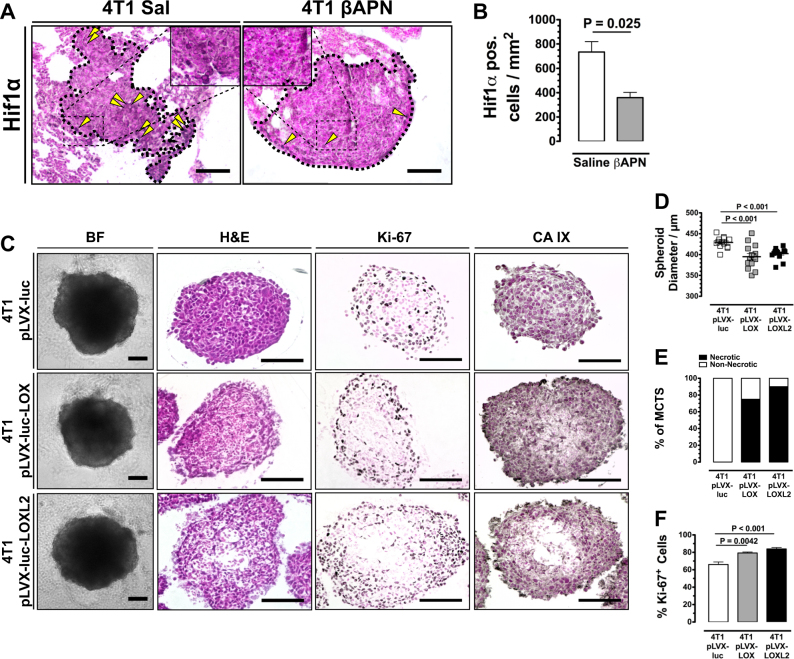
Reduced supply is a direct effect of **a** Hif1α staining in non-vascularized spontaneous lung metastases (outlined) arising in mice implanted with 4T1 tumors. Yellow arrowheads: cells with nuclear Hif1α immunreactivity. **b** Quantification of tumor cells in non-vascularized 4T1 lung metastases with nuclear Hif1α staining (*n* = 4). **c** Histological and immunohistological analysis of MCTS generated from LOX/LOXL2 OE 4T1 cells. **d** Size distribution of 4T1 control and 4T1 LOX/LOX2 OE MCTS after 6 days of cultivation (*n* = 13). **e** Quantification of central necrosis in 4T1 control and 4T1 LOX/LOX2 OE MCTS (*n* = 8). **f** Quantification of Ki-6^+^ cells in 4T1 control and 4T1 LOX/LOX2 OE MCTS (*n* = 8). Error bars: ±SEM. SB = 100 µm. * indicates statistical significance vs. control, *P < 0.05, **P < 0.01

The results so far indicated that high lysyl oxidase activity by increasing ECM cross-linking and stabilizing collagen content deprives the tumor of necessary supply, which also delimits transport of drugs into the tumor. However, it has also been reported that lysyl oxidase activity and collagen cross-linking has a strong effect on tumor angiogenesis [22, 26]. The vascular status, that is vessel density and vascular integrity, strongly influences transport of drugs and oxygen. To distinguish between immediate effects of the increasingly dense and rigid ECM and secondary effects via vascular changes on tumor supply, we evaluated the hypoxic response in spontaneous lung metastases arising in 4T1-implanted mice. By narrowing the evaluation process on small non-vascularized metastases, any vascular influence on the oxygenation status of the metastases was excluded. Developing metastases in mice showed significant lower immune reactivity for Hif1α after treatment with βAPN for 12 days (Fig. 4a, b). Multicellular tumor spheroids (MCTS) generated from LOX or LOXL2 overexpressing 4T1 cells were reduced in size (Fig. 4c, d). The reduced growth can be attributed to a reduced diffusion as the LOX/LOXL2 OE MCTS were characterized by a necrotic, undersupplied core—which was absent from control MCTS—and increased staining for CAIX (Fig. 4c, e). A proliferative effect can be excluded as the non-necrotic rim of the MCTS showed increased staining for KI-67 (Fig. 4c, f). These results demonstrate that the increased supply with nutrients and oxygen observed in tumors after LOX(L) inhibition is a direct effect of the improved diffusivity of the less crosslinked and stabilized collagen matrix and not a secondary effect of altered vascularization.

### Lysyl oxidase overexpression renders tumors resistant to chemotherapy

**Fig. 5:**
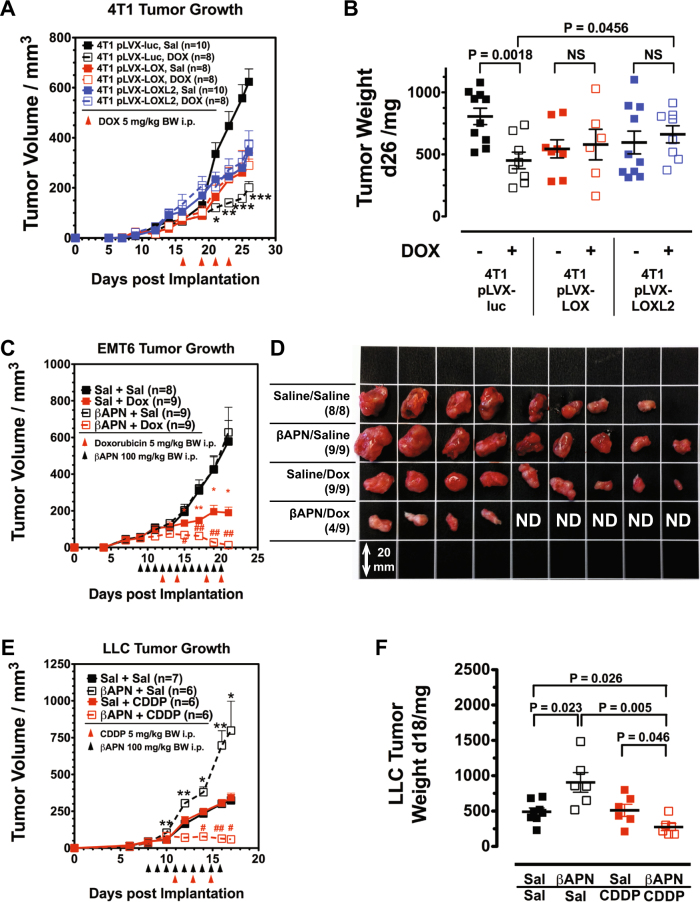
Lysyl oxidase activity impairs response toward cytotoxic chemotherapeutics in primary tumors and lung metastases. **a** Treatment of established 4T1 control and 4T1 LOX/LOX2 OE BCAs with DOX. **b** Weight of treated 4T1 control and 4T1 LOX/LOX2 OE BCAs 26 days after implantation. **c** Treatment of established EMT6 BCAs with βAPN and DOX. Although βAPN did not reduce tumor growth of EMT6 BCAs, it increased response to DOX. **d** Size distribution of EMT6 BCAs after treatment. **e** Treatment of established LLC tumors with βAPN and CDDP. **f** Weight of treated LLC tumors 18 days after implantation. Combination of βAPN and CDDP significantly reduced tumor size. Error bars: ±SEM. * indicates statistical significance vs. control, # indicates statistical significance of combination treatment group vs. both single treatment groups: *^,^^#^*P* < 0.05, **^,^^ ##^*P* < 0.01, ***P < 0.001

To verify that lysyl oxidase activity confers therapeutic resistance, 4T1 tumors overexpressing either LOX or LOXL2 were treated with DOX, a chemotherapeutic that showed good response in 4T1 tumors [21]. Overexpression of LOX/LOXL2 increased tumor establishment and initial growth of the 4T1 tumors (Fig. 5a). Treatment with DOX was started during this initially growth-promoting stage and continued into the second phase when the increasingly dense ECM inhibited tumor supply and growth. While control tumors (4T1-pLVX-luc) reacted to DOX with a significant reduction in growth, LOX/LOXL2 OE tumors were completely resistant to the chemotherapeutic (Fig. 5b).

Improved homogeneity in drug distribution and increased overall accumulation of drugs was observed after lysyl oxidase inhibition. This indicates the potential of lysyl oxidase inhibition as an auxiliary treatment to improve therapeutic response to cytotoxic drugs. To test this, a combination treatment regimen with βAPN and chemotherapeutic drugs was designed: fully established EMT6 and LLC tumors were treated with an initially βAPN phase, to improve tissue diffusivity, before either DOX or cisplatin (CDDP) was administered. In the EMT6 model, βAPN again did not affect tumor growth. However, it significantly increased response toward DOX (Fig. 5c). After a 12-day treatment period, tumors in five of nine animals were no longer detectable (Fig. 5d). In the LLC model, we evaluated response toward lysyl oxidase inhibition plus CDDP (Fig. 5e, f). Interestingly, both agents as stand-alone therapies were ineffective at the chosen dosages (100 mg/kg BW qd, and 5 mg/kg BW q2d, respectively): while CDDP did not affect tumor size, βAPN strongly increased the growth rate of the LLC tumors. However, combining both ineffective drugs had a significant therapeutic effect.

**Fig. 6:**
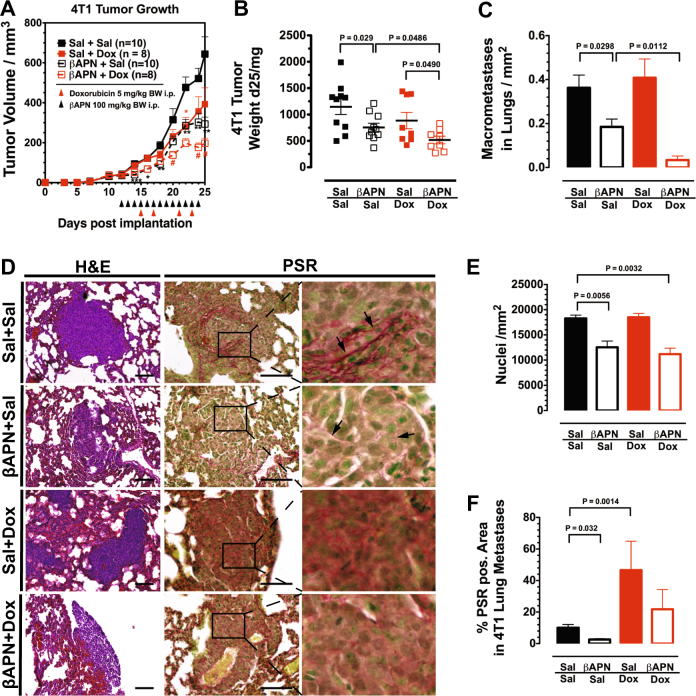
LOX(L) inhibition improves treatment efficacy in metastatic disease. **a** Treatment of established 4T1 BCAs with βAPN and/or DOX. **b** Weight of treated 4T1 BCAs 25 days after implantation. Combination of βAPN and DOX significantly reduced tumor size. **c** Quantification of metastatic nodules in the lungs of 4T1-implanted mice after a 14 days treatment course with βAPN and/or DOX. **d** Histological evaluation of metastases in the lungs of 4T1-implanted mice after a 14 days treatment course with βAPN and/or DOX. H&E and PSR staining of lung tissue. The metastases appear disintegrated after βAPN treatment. Collagen fibrils (arrows) are strongly reduced in size and quantity. **e** Cell density in metastases. Nuclei were quantified in H&E-stained sections (*n* = 6). **f** Quantification of PSR staining in metastases (*n* = 6). SB: 100 µm. Error bars: ±SEM. * indicates statistical significance vs. control, # indicates statistical significance of combination treatment group vs. both single treatment groups: *^,#^*P* < 0.05, **^,##^*P* < 0.01, ***P < 0.001

To evaluate the effect of a combination treatment on metastatic disease, we treated established βAPN-responsive and highly metastatic 4T1 tumors with βAPN and DOX. The treatment was started 12 days after orthotopic implantation of 4T1 tumors into the mammary fat pad of the animals, a time point where metastatic seeding can be observed in all implanted animals [27]. In this tumor model, βAPN treatment effectively inhibited tumor growth and DOX had also a considerable anti-tumor effect. Combination of the two similarly effective drugs resulted in a further, significant reduction in tumor growth (Fig. 6a, b). Interestingly, when the number of metastatic nodules in the lungs of the treated animals was evaluated at the end of the 14-day treatment period, it turned out that DOX/βAPN combination lowered the rate of metastatic incidents by 90% (9.4 ± 5.0% compared to control-treated animals, Fig. 6c). In contrast, DOX alone was ineffective in reducing the number of macrometastases, while βAPN alone showed a moderate although statistically significant effect (50.9 ± 10.0% macrometastases vs. control). As stand-alone treatment, both agents reduced the size of the metastases (Supplementary Figure S3). The effect was significantly stronger in animals receiving the combination treatment in which larger metastases above 60 µm^2^ were completely absent—in control-treated animals, metastases reached 507.5 µm^2^, in DOX and βAPN-treated animals 147 µm^2^ and 142 µm^2^, respectively. The metastases in βAPN-treated mice appeared loosely packed, without the dense structure observed in untreated animals (Fig. 6d). This was evident by the strongly reduced cell density in the metastases (Fig. 6e). Picosirius red (PSR) staining showed a nearly complete absence of collagen fibrils after βAPN treatment (Fig. 6d, f). On the other hand, DOX by itself massively increased collagen deposition. However, the staining appeared more diffuse, without clear fibrils. Metastases in animals receiving βAPN/DOX combination treatment retained the loose appearance caused by LOX(L) inhibition but also showed the increased collagen content in response to the DOX treatment. Thus, LOX(L) inhibition interfered with the ability of the nascent metastases to form a protective environment characterized by a dense cell packing and supportive barrier of fibrillar collagen. Consequently, the chemotherapeutic agent that was not able to reduce the absolute number of metastases could effectively eradicate the remaining, immature metastases.

### Tumor hypoxia intolerance and reliance on FAK signaling controls response toward LOX(L) inhibition

**Fig. 7:**
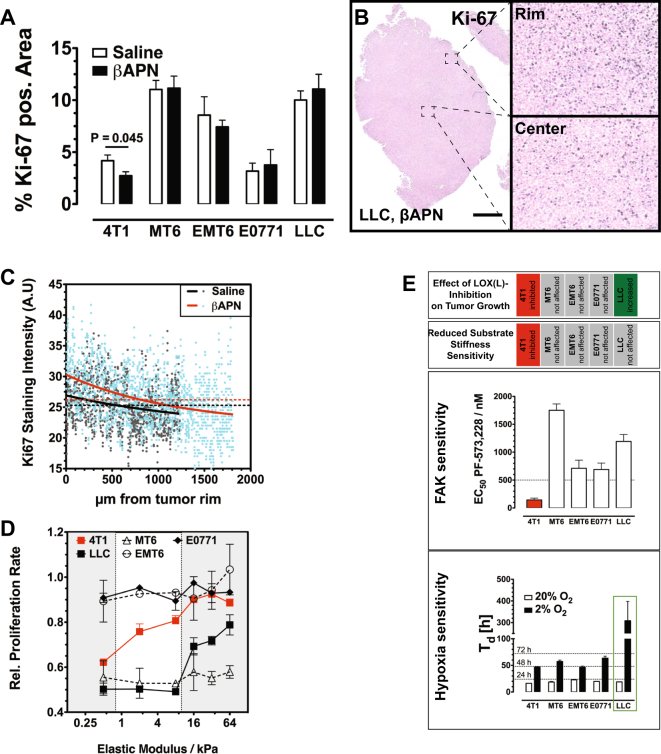
Hypoxia sensitivity and reliance on FAK signaling controls response toward LOX(L)-inhibition. **a** IHC staining for Ki-67 shows that changes in proliferating cell density correlate with the effects on growth rate by LOX(L) inhibition (*n* = 6–10). **b** Regional differences in Ki-67 staining in LLC tumors. In the central region of the tumor density of proliferating cells is significantly lower than in the highly proliferative rim region (SB = 1000 µm). **c** Spatial resolution of Ki-67 staining intensity in βAPN-treated and control LLC tumors, following trajectories from the tumors’ outer margins to the center (*n* = 4). **d** Relative proliferation rate of murine tumor cells on substrates with variable stiffness (*n* = 3). **e** Effect of FAK inhibition and reduced oxygen supply on tumor cell proliferation and viability in cell culture. EC_50_ values of FAK inhibitor PF-573,228 in various cell lines. Selected threshold value defining sensitivity to PF-573,228 (EC_50_ < 500 nM) is indicated. (*n* = 3). Doubling time (*T*_d_) of tumor cells at reduced oxygen levels (2% O_2_) (*n* = 5). Error bars: ±SEM. * indicates statistical significance vs. control, *P < 0.05, **P < 0.01, ***P < 0.001

LOX(L) inhibition by itself affected tumor growth in the five tested models very differently, which has significant implication for the clinical use of LOX(L) inhibitors as auxilliary agents. To investigate why growth was affected so differently, we first examined treated tumors for changes in proliferation and apoptosis. Staining of tumor sections for KI-67 revealed a reduced density of proliferative cells in the responsive 4T1 tumors, while in the non-responsive MT6, EMT6, and E0771 tumors proliferation rate was not changed (Fig. 7a). Intriguingly, in the inverse-responsive LLC tumors, the proliferative zone at the tumor rim was significantly enlarged (Fig. 7b, c). Staining for cleaved caspase-3 (ClCasp-3) in the responsive 4T1 tumors did not show any differences between the treated and control tumors, excluding an additional apoptotic effect (Supplementary Figure S4). Lysyl oxidase inhibition also influenced proliferation of cells in MCTS (Supplementary Figure S5). The results obtained in the in vitro system correlated with the in vivo findings: 4T1 MCTS showed reduced proliferation rates upon βAPN treatment, while in LLC MCTS the percentage of proliferating cells was increased. In the in vitro system, effects of infiltrating TACs that might influence tumor proliferation can be excluded.

After having verified that lysyl oxidase activity depending on the tumor can affect proliferation and tumor growth in both ways, either negatively or positively, we now examined how these oppositional effects could be explained. We hypothesized that lysyl oxidase activity could affect tumor growth in two converse ways: on one hand, it has been shown that increased tissue rigidity, e.g., as a consequence of lysyl oxidase activity, can increase tumor cell proliferation [28, 29]. On the other hand, our results so far demonstrated that increased ECM cross-linking universally reduces diffusion and thereby supply with oxygen and nutrients, which should restrict proliferation. Differential responsiveness toward tissue stiffness on one hand and sensitivity nutritional deprivation on the other would explain the varying effects of lysyl oxidase inhibition on the growth of different tumors. We first tested the effect of variation of substrate stiffness on the proliferation rate in vitro, by seeding the five cell lines used to generate tumors on substrates with varying rigidity (Fig. 7d). Growth of E0771 and EMT6 was barely affected by variations in substrate rigidity, while 4T1, MT6, and LLC cells were sensitive to reduction in substrate stiffness. However, 4T1 cells were the only cell line reacting to changes in substrate stiffness within the critical range of 0.8–10 kPa, the range of the elastic modulus (Fig. 7e) commonly observed in solid tumors. βAPN treatment also causes changes of the elastic modulus in this range [30–32]. Growth of the two other sensitive cell lines (LLC and MT6) was already reduced to a minimum at *E* values higher than 10 kPa and was no longer affected by further softening of the substrate within the physiologically relevant range. As it has been shown previously that lysyl oxidase-catalyzed ECM cross-linking increases integrin signaling and subsequently proliferation in a FAK-dependent way [28], we tested sensitivity of the cell lines toward the FAK inhibitor PF-573228 [33]. 4T1 cells were the only cell line responsive toward PF-573228 (Fig. 7e). To examine dependency of the tumor cells on oxygen supply, their proliferation rates were evaluated at 20 and 2% oxygen levels. LLC cells were the cell line most drastically affected in their proliferation by reducing oxygen levels to 2% (Fig. 7e). Thus, in line with our hypothesis, βAPN treatment reduced growth only in tumors formed by the sole cell line sensitive toward substrate rigidity and FAK inhibition. On the other hand, improved oxygenation after LOX(L) targeting solely enhanced growth of the tumors derived from the most hypoxia-sensitive cells.

**Fig. 8:**
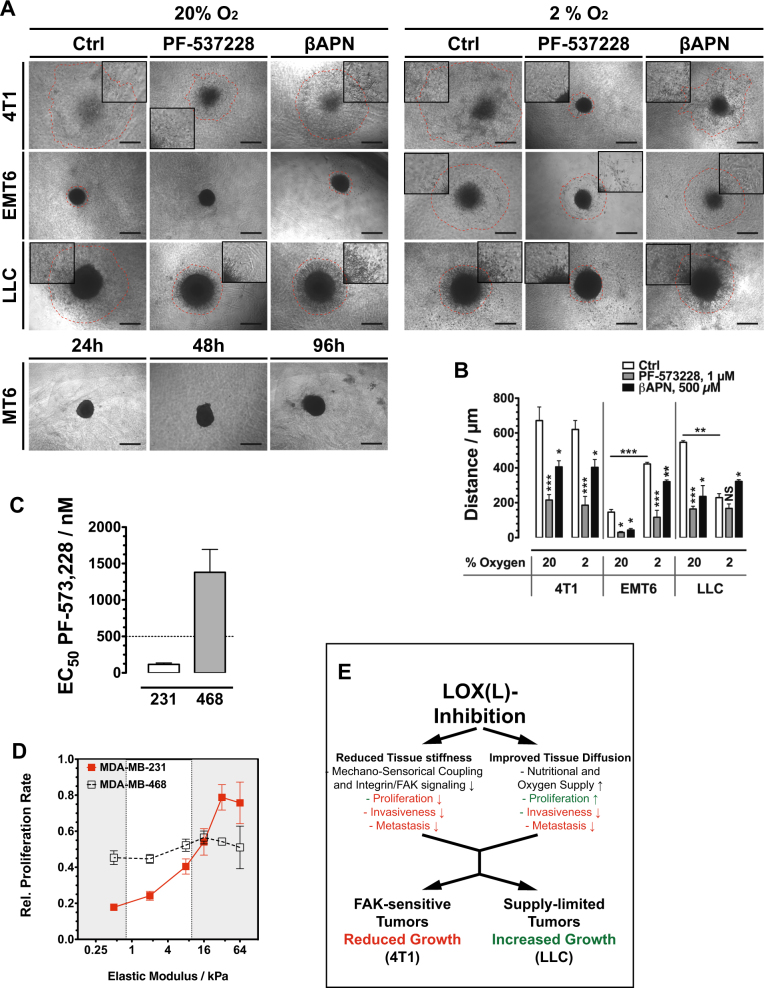
**a** Invasive behavior of tumor cells in a MCTS collagen-embedding experiment. MCTS generated from different cell lines were embedded in collagen I and treated with FAK and LOX inhibitors. Dashed red lines mark the front of migrated cells after 96 h. **b** Quantification of the effect of FAK and LOX(L) inhibition on the invasive behavior in an MCTS collagen-embedding experiment (*n* = 4). **c** EC50 values of FAK inhibitor PF-573,228 in human breast cancer cell lines MDA-MB-231 and -468. MDA-MB-231 cells are sensitive (EC50 < 500 nM) MDA-MB-468 insensitive to PF-573,228 (*n* = 3). **d** Relative proliferation rate of human breast cancer cell lines MDA-MB-231 and -468 on substrates with variable stiffness (*n* = 3). **e** Schematic summary of how FAK and hypoxia sensitivity regulates response of tumors to LOX(L) inhibition. Error bars: ±SEM. * indicates statistical significance vs. control, *P < 0.05, **P < 0.01, ***P < 0.001

FAK signaling is also involved in cell migration and invasiveness. By generating multicellular tumor spheroids from the studied cell lines and embedding those in a collagen matrix we were able to monitor cell invasion into the surrounding matrix (Fig. 8a). At 20% oxygen, all three tested cell lines reacted sensitive toward FAK and lysyl oxidase inhibition, with reduced invasiveness (Fig. 8a, b). FAK inhibition also affected invasiveness of cell lines that were insensitive with respect to proliferation. FAK signaling directs cell motility via Rho/Rock signaling while proliferation is influenced via MEK and Erk phosphorylation. At reduced oxygen (2%), LLC cells showed strongly reduced invasiveness that could not further be inhibited by PF-573228, but was increased by lysyl oxidase inhibition, indicating that at the reduced oxygen levels invasive behavior was largely limited by decreased proliferation. Lysyl oxidase inhibition improved oxygenation leading consecutively to increased proliferation and invasive potential.

We further investigated the different sensitivity toward FAK inhibition, which correlated with dependency on ECM cross-linking. We evaluated expression of FAK, uPAR, EGFR, and Merlin/NF2, a protein linking the cytoskeleton, to the cell membrane that has been recently reported to confer resistance to FAK inhibition [34, 35]. Expression of neither of these genes was substantially different in the sensitive 4T1 cells compared to the non-sensitive cell lines (Supplementary Figure S6).

To finally test the hypothesis that dependence on substrate stiffness for proliferation and sensitivity toward FAK inhibition are correlated, we evaluated proliferation rates of two human cell lines on variously rigid substrates. MDA-MB-231 and MDA-MB-438 were reported FAK inhibition sensitive and insensitive, respectively [34]. Indeed, did the two cell lines display substantial differences under treatment with PF-573228, as growth was inhibited with an EC_50_ of 117.2 ± 19.2 nM (MDA-MB-231) and 1379 ± 315 nM (MDA-MB-468) (Fig. 8c). By plating cells on soft substrate plates and following proliferation, MDA-MB-231 displayed a significant dependency on more rigid substrates, with a strong decline in proliferation rate in the critical range of 10–0.5 kPa (Fig. 8d). The FAK-insensitive MDA-MB-468 cells also did not response with significant proliferation changes to a reduction in substrate stiffness.

These results demonstrate a delicate balance between two effects of lysyl oxidase activity that influence proliferation in the tumor: increased tissue stiffness has a pro-proliferative effect on FAK-sensitive tumors while the reduced interstitial transport limits proliferation in supply limited tumors (Fig. 8e).

## Discussion

Our approach to systematically profile the expression of ECM molecules in chemoresistant tumors identified a signature of high collagen expression and increased stabilization as a marker of intrinsic resistance. In subsequent modeling experiments, we were able to discover that increased collagen deposition is not only a marker, but a direct proximate cause for resistance and to elucidate the underlying mechanisms of reduced supply, increased hypoxic and metabolic stress, further aberrant angiogenesis, enhanced inflammation, and impeded drug distribution.

We systematically evaluated expression data from cancer patients with the explicit aim to identify ECM components conferring chemoresistance, and importantly druggable targets that can be used to inhibit the build-up of these components in the tumor microenvironment. The data sets used for this analysis were generated with material from patients suffering from different malignancies—with a strong bias toward breast cancer. The patient groups received various regimens of chemotherapeutic-based treatment and criteria for a classification as resistant and sensitive undoubtedly also varied between the different teams that assembled the data. No actions were taken to assimilate data or statements about drug response between the sets, but the data sets were evaluated independently side by side, and results were compared to identify broad, comprehensive patterns. This approach is unquestionably unsuitable to discover genes with subtle expression differences between resistant and sensitive tumors, and targets that are linked to resistance only in certain tumors or that convey resistance only to certain drug classes. The approach was deliberately designed to pick the low hanging fruits only, with the hope that whatever was lost in terms of sensitivity was gained in robustness. To further solidify the approach, a first focus was on the analysis of entire protein families and groups of proteins with related function. Laminin, proteoglycan, and collagen expression stood out as correlated with resistance, while interestingly others like elastins and hyaluronan-related proteins appear not to be altered. Collagens were selected for further analysis as their complex, but well-understood processing, maturation, and maintenance involves many druggable enzymes [36].

A central consideration was that diverse mechanisms could lead to intrinsic resistance toward therapy, and that resistant tumors can be subdivided into different classes, according to the individual mechanism that contributes most strongly to chemoprotection. Following this hypothesis, a subgroup of resistant tumors was identified that showed strong expression of several collagens, among them the most abundant fibrillar collagens, collagen type I and II. Interestingly, when we screened within the expression profile of these collagen-high resistant tumors for druggable targets, only lysyl oxidases were consistently upregulated. Prolyl-4-hydroxylases (P4H), prolyl-3-hydroxylases (P3H), and BMP1 were only upregulated in some data sets. Of the PLOD family, we found only PLOD2 elevated in one data set. PLOD2 has been shown to contribute to metastatic behavior via ECM modification leading to increased hypoxia, an effect very similar to the mechanisms caused by LOX(L) [9, 37].

We validated the chemoprotective role of lysyl oxidases in several tumor models. Consistently, inhibition of total LOX(L) activity improved oxygenation, overall drug uptake, and homogenous drug distribution within the treated tumors. Ectopic expression of the two lysyl oxidase family members LOX and LOXL2 led to the opposite effect. LOX(L) inhibition significantly increased response to two tested chemotherapeutics with distinct mechanisms of action: DOX, a topoisomerase II inhibitor and CDDP that resembles an alkylating agent. Thus, the sensitizing effect of βAPN treatment is not restricted to drugs utilizing a specific cytotoxic mechanism. Strong LOX/LOXL2 overexpression in collagen-rich 4T1 tumors rendered them completely resistant to chemotherapy, again indicating that not off-target effects of βAPN, but its ability to block LOX(L) function is crucial for its augmenting activity. This finding has implications how LOX(L) inhibitors should be tested in the patients. Recently, the failure of an anti-LOXL2 antibody (simtuzumab) to improve PFS in two phase II trials was reported [38, 39]. Although simtuzumab was tested in combination with chemotherapy—a FOLFIRI regiment in colorectal cancer and gemcitabin in metastatic pancreatic carcinoma—the two studies were not designed to provide for a potential drug delivery improvement after LOXL2 targeting, rather the studies were based on the previous preclinical findings that LOXL2 inhibition by itself affects tumor progression. Scheduling that takes into account time for ECM remodeling after LOXL2 inhibition might have improved results, notwithstanding the challenging situation to improve treatment options in strongly progressed and pretreated disease. Moreover, given the functional redundancy of the individual LOX family members and their overall elevated expression in tumors, in all likelihood targeting just one family members will not result in a sufficient remodeling of the ECM. Small-molecule inhibitors of lysyl oxidase activity or the targeting of upstream effectors of LOX(L) expression, like the TGFβ pathway, might be more promising [40, 41].

Previous studies have reported a solid and consistent anti-tumor effect of LOX(L) inhibition in a variety of different tumor models [23–25]. Baker et al. have demonstrated that the proliferative effect of lysyl oxidases is caused by increased tissue stiffness and subsequently enhanced FAK signaling [28]. However, proliferative response to changes in substrate stiffness varies strongly between different tumor cells [29]. Moreover, the vast majority of tumors (78%) is non-responsive to FAK inhibition [34]. This suggests that LOX(L) inhibition should not have a general anti-proliferative effect on tumors. In line with this conclusion, we found that response of implanted tumors to LOX(L) inhibition was closely mirrored by the effect that reduction in substrate stiffness or FAK inhibition had on the proliferation rate of the respective tumor cells in vitro. Only one of the five cell lines tested (4T1) formed tumors that responded to LOX(L) inhibition with reduced growth. 4T1 cells were also the only cell line sensitive to changes in substrate stiffness and to FAK inhibition. On the other hand, tumors whose growth were evidently limited by supply (LLC) responded with increased growth to LOX(L) inhibition. The delicate balance between pro-proliferation FAK signaling and reduced supply was further demonstrated by overexpression of LOX and LOXL2 in 4T1 tumors: the overexpressing tumors grew faster in the beginning until support for this rapid expansion was limited by the inhibited diffusion through the dense matrix. 4T1 tumors express high levels of collagen I and II, but are low in LOX(L) expression [42]. Thus, the ectopic expression was able to effectively transform the matrix in these tumors, leading to an even higher collagen content, and a denser fibrillar network.

Our data excluded vascular effects. In line with previous findings that reported reduced angiogenesis after LOX(L) inhibition [22, 26], overexpression of LOX family members increased perfused vessel density. In addition, supply-limiting effects were observed in systems—MCTS and non-vascularized metastases—in which vascular effects can be excluded. In contrast, Le Calve et al. reported a strong increase in vascular collapse after LOXL2 application, which they considered causal for reduced drug supply and response [18]. The fact that they applied LOXL2 by systemic injection rather than by ectopic overexpression in situ might explain why their results are in conflict with other findings. The delivery route via the vasculature of course would impact the vicinity of blood vessels as the strongest. As lysyl oxidases also act on collagen IV, the main component of the vascular basal lamina, this might significantly affect the integrity of the blood vessels.

Given the narrow therapeutic windows of most anti-cancer drugs, methods to increase transport into the tumor and importantly homogeneity of distribution of therapeutic agents could at once enhance the efficacy of the full array of cancer therapeutics at our disposal. Our results clearly demonstrate the potential of targeting the tumor ECM as a way to simultaneously ameliorate the malignant tumor microenvironment, and to improve delivery of therapeutic agents. This could open the path to a strategic approach in the systemic management of malignant diseases, aiming to first transfer the tumor in a less malignant and importantly more vulnerable state before eradicating it with cytotoxic treatment.

## Experimental procedures

### General

If not otherwise indicated, chemicals were purchased from Sigma-Aldrich (Munich, Germany) or Carl Roth (Karlsruhe, Germany). Protein concentrations were determined with the Pierce BCA Kit (Thermo Fisher, Rockford, IL), using a 30-min incubation time at 60 °C.

### Microarray analysis

Data sets were downloaded from NCBI’s Gene Expression Omnibus (http://www.ncbi.nlm.nih.gov/geo/). Analyses were performed using BRB-ArrayTools developed by Dr. Richard Simon and the BRB-ArrayTools Development Team (http://brb.nci.nih.gov/BRB-ArrayTools/index.html) [43] and visualized with ClustalX and Treeview. Gene lists for cluster analysis were compiled using BRB-Array’s GO retrieval utility or manually assembled from data in the literature [44]. For differences in gene expression between different array groups (classes), class comparison was performed with a significance threshold of 0.05.

### Cell culture

MT6 (CRL-2805), 4T1 (CRL-2539), LLC (CRL-1642), MDA-MB-231, and MDA-MB-468 cells were obtained from ATCC. EMT6 cells have been purchased from NCI Tumor Repository (http://ncifrederick.cancer.gov/Services/NcifRepositories.aspx). E0771 cells have been purchased from Tebu-Bio (Offenbach, Germany). All tumor cells were maintained in DMEM (Gibco) with 10% FBS and penicillin/streptomycin at 37 °C, 5% CO_2_, and tested at least annually for mycoplasma contamination.

### Production of lentiviral particles and generation of 4T1 cell lines expressing hLOX and hLOXL2

The entire CDS of both hLOX and hLOXL2 CDS, including the signal peptide, was amplified from HUVEC cDNA. The amplified DNA was cloned behind the IRES sequence into the lentiviral vector pLVX-luc-IRES-puro (Clontech, Mountain View, CA). Lentiviral particles were generated in HEK 293T cells by co-transfection with the pCMV-dR8.9 and pCMV-VSV-G [45] (both plasmids were obtained from Addgene, Cambridge, MA), using a standard CaCl_2_-based transfection method. Supernatant was used to transfect 4T1 tumor cells. Stable cells selected with puromycin (5 µg/mL). To generate a control cell line, 4T1 cells were transfected with lentiviral particles produced in HEK 293T cells using the pLVX-luc-IRES-puro plasmid.

### Multicellular tumor spheroids

Tumor spheroids were generated by the liquid overlay technique, using the protocol from Walser et al. with slight modifications [46]. Wells of a 96-well culture plate were coated with 45 µL of 1.2% agarose. After the agarose had solidified, 2000 tumor cells were seeded in 200 µL medium (supplemented with 500 µM βAPN where applicable) on top of the coating. Cells were incubated at 37 °C, 5% CO_2_ for 6 days with an exchange of 50% media volume on day 4.

### Tumor models and treatment

All experiments involving animals were reviewed and approved by the Institutional Animal Care and Use Committee at MSKCC or by the Regional Administration of Unterfranken, Würzburg. The experiments were performed in accordance with relevant guidelines and regulations.

#### Tumor engraftment

MT6 fibrosarcomas (1 × 10^6^ cells in PBS) and LLC (1 × 10^6^ cells in matrigel) tumors were generated by subcutaneous injection in the dorsal region of female C57Bl/6J mice. E0771 (1 × 10^6^ cells in PBS) breast adenocarcinomas were generated by injection of cells into the inguinal mammary fat pad of female C57Bl/6J mice. 4T1 (1 × 10^5^ cells in PBS) breast adenocarcinomas and EMT6 (1 × 10^6^ cells in PBS) breast adenocarcinomas were generated by injection of cells into the inguinal mammary fat pad of female Balb/c mice. Balb/c mice were purchased from Charles River, Sulzfeld Germany, Balb/c and C57Bl/6J from Jackson Labs, Bar Harbor, ME. Sample sizes to detect the pre-specified effects were calculated using the software G*power [47].

All animals in the individual experiments were of the same age and sex. For each experiment, tumor-bearing mice were randomly assigned to the different treatment groups just prior to the start of treatment. In treatment studies where tumor growth was a critical outcome assessment of tumor size was performed blinded, by a second researcher.

#### Exclusion of data

Animals that never developed tumors due to take rate lower than 100% were excluded from the studies. All data from animals that died or had to be killed prior to the scheduled termination of the experiment was excluded.

#### Tumor treatment

Three-aminopropionitrile fumarate was administered at 100 mg/kg or 30 mg/kg BW in 0.9% NaCl by daily intra peritoneal injection. Control substance was Na-fumarate in 0.9% NaCl. Doxorubicin (DOX) and cisplatin (CDDP) were administered i.p at 5 mg/kg BW on indicated days. Control substance for DOX/CDDP was 0.9% NaCl.

Tumor growth was followed by measuring perpendicular diameters of the tumors with a vernier calliper. Tumor volume was calculated using the equation *V* = *π*/6 × l × *w*^2^. In addition, tumors were excised post mortem and weighted. Only tumors that could be excised completely without additional invaded tissue were used for weight measurements.

### IHC and IF staining of tumor sections

H&E, picrosirus red, IHC, and IF staining were performed using standard techniques on formalin-fixed paraffin-embedded sections. Tissues for quantitative evaluation were processed in parallel. For quantification, whole tissue sections were imaged on a Keyence BD microscope with an automated stage. The whole virtual slide was used for quantification using the ImageJ software package (rsbweb.nih.gov/ij/).

Quantification of PSR staining was performed using ImageJ. RGB (Red,Green,Blue) images were split in the three-color channels. The green channel was used for quantification of the relative area that displayed a signal above a certain, constant threshold.

Antibodies used for IHC/IF or WB: cleaved caspase-3 (Cell Signaling Technology Cat# 9661, RRID:AB_2341188), Carbonic Anhydrase IX (Santa Cruz Biotechnology Cat# sc-25599, RRID:AB_2066539)), Hif1α (Novus Cat# NB100–131H, RRID:AB_1108863), CD31 (Santa Cruz Biotechnology Cat# sc-28188, RRID:AB_2267979), CD34 (Abcam Cat# ab8158, RRID:AB_306316), Collagen IV (Bio-Rad / AbD Serotec Cat# 2150-1470, RRID:AB_2082660), Ki-67 (Abcam Cat# ab16667 RRID:AB_302459), LOX (IMGENEX Cat# IMG-6442A RRID:AB_1930256), LOXL2 (Biorbyt Cat# orb41134 RRID:AB_10987961), β-Actin (Santa Cruz Biotechnology Cat# sc-1615 RRID:AB_630835).

### Hoechst distribution, lectin vessel staining, and 3D image evaluation

To monitor intratumoral distribution of drugs, 50 µL of Hoechst 33342 stock solution (Sigma, 20 mg/mL in 0.9% NaCl) and 50 µL of Alexa 647-labeled Isolectin GS-B4 (Life Technologies, Darmstadt, Germany. 500 µg/mL in 0.9% NaCl) were injected i.v. into tumor-bearing mice, 20 min before killing the animal. Tumors were removed and flash frozen in OCT (Sakura Finetek Torrance, CA).

For Hoechst 33342 tissue penetration and 3D vessel evaluation, tissue was cut on a cryotom to 200 µm slices and mounted on glass slides. Z-stacks were acquired by confocal (Nikon A2, ×20 objective) imaging by excitation with a 405 and 647 nm laser line. Tissue penetration was measured as the maximal distance from the vessel surface (by Alexa 647 staining) that Hoechst 33342 staining was present using ImageJ. For this purpose, the acquired z-stacks were evaluated at the same tissue depth for isolated, longitudinal cut blood vessels. The maximal distance of Hoechst 33342 staining was measured perpendicular to both sides of each blood vessel, the arithmetic mean of the two values was used. Each blood vessel was evaluated at several positions. At least 10 vessels per stack, and four stacks per biological sample were evaluated.

3D vessel evaluation was done using the ImageJ software package (rsbweb.nih.gov/ij/) or its Fiji distribution (http://fiji.sc/wiki/index.php/Fiji) with additional plugins: Skeletonize 3D (http://imagejdocu.tudor.lu/doku.php?id=plugin:morphology:skeletonize3d:start) [48], Tubeness (http://www.longair.net/edinburgh/imagej/tubeness/). For vessel ramification analysis, binary stack images were converted with the skeletonize plugin and evaluated for branching points. Vessel surface area was evaluated with the tubeness plugin.

### Biodistribution of doxorubicin

For biodistribution studies, a bolus of 100 µg doxorubicin in 0.9% NaCl was injected intra peritoneal on specified days to doxorubicin naive animals. Mice were killed 2 h post injection when doxorubicin could be expected to be cleared from the blood stream [49]. Tissue samples were flash frozen and stored at −80 °C until extraction. The method described by Laginha et al. was used with slight modifications [50]. In brief, tissue samples were homogenized by sonification in nine parts (v/w) water. Aliquot of 200 µL homogenate were combined with 50 µL 10% Triton X-100 (v/v) and 750 µL 0.75 N HCl in 2-propanol. The mixture was vortexed briefly and extracted for 12 h at −20 °C. Samples were again vortexed at r.t. and cleared by centrifugation (20 min, 4 °C, 20,000 × *g*). Fluorescence was read (Ex.: 470 nm, Em.: 590 nm) in a microplate reader and corrected against extracts from tissue samples of non-treated animals. A standard curve was established by adding defined amounts of doxorubicin/doxil to homogenates of non-treated tissue samples prior to extraction.

### ECM extraction

Extracellular matrix proteins were extracted from tumor tissue using a modified protocol from Kleinman et al. [51]. In brief, tumors (size 300–500 mm^3^) were excised, weighted, snap frozen, and stored at −80 °C until further work-up. The tumors were homogenized in 2 mL/g WW high salt extraction buffer (HSEB, 3.4 M NaCl, 50 mM Tris HCl, 4 mM EDTA, pH 7.4) on ice with a tissue homogenizer (UltraTurax, IKA, Staufen, Germany). Non-soluble material, including ECM proteins, was pelleted by ultracentrifugation (100.000 × *g*, 4 °C, 30 min). This HSEB extraction was repeated once supernatants were collected for western analysis. The pellet was washed with water and PBS, and finally re-suspended in PBS.

For urea extraction, the HSEB non-soluble pellet was re-suspended in 1.8 mL/g (starting material) of a urea extraction buffer (UEB, 2M urea, 150 mM NaCl, 50 mM Tris HCl, 4 mM EDTA, pH 7.4) briefly homogenized and extracted overnight at 4 °C. Still non-soluble material was again pelleted by ultracentrifugation (26.000 × *g*, 1 h 4 °C). The UEB supernatants were dialyzed against a low salt buffer (150 mM NaCl, 50 mM Tris HCl, 4 mM EDTA, pH 7.4) for 48 h at 4 °C with two buffer changes. Protein content was determined with the BCA Assay Kit.

To all extraction buffers, complete proteinase inhibitor cocktail (Roche Diagnostics, Mannheim, Germany) was added.

### Collagen quantification

The relative collagen content of tissues was measured using a method reported by Lopez de Leon and Rojkind with slight modifications [52]. In short tissue, sections were deparaffinized and rehydrated before incubation with a solution of 0.1% (w/v) direct red 80 and 0.1% (w/v) fast green FSF in water saturated with picric acid for 30 min at r.t.. The stained material was washed excessively with water (6 × 15 mL) before being extracted with 1 mL of 0.1 N NaOH in water/methanol 1:1 (v/v). Absorbance of the resulting solution was read in a spectrophotometer at 530 nm (direct red 80) and 630 nm (fast green). A compensation curve for the fast green absorbance at 530 nm was established beforehand and used to correct direct red 80 reading at this wavelength.

### Collagen cross-linking analysis

ECM from βAPN-treated and control tumors was obtained by high salt extraction of cellular components. The insoluble ECM was re-suspended in water and used to coat glass slides (angiogenesis µ-slides, Ibidi, Martinsried, Germany) at µg/well. Interferences reflection Images were acquired as z-stacks (30 slides, z-distance: 1.0 µm) on a Nikon A1 microscope in reflection mode using a ×60 oil immersion objective and a 647 nm laser following a published protocol [53]. Identifiable collagen fibers in optical fields were manually counted.

### Transwell ECM drug penetration assay

The membranes of transwell inserts (24-well MWD format, 33 mm^2^ membrane area, Costar, Cölbe, Germany) were coated with 3 µg/mm^2^ of the respective ECM extract or protein by adding the protein suspension in 50 µL of buffer and letting the membranes air dry overnight. ECM was reconstituted by adding 150 µL of PBS to the upper chamber of the transwell and incubation for 1 h. Aliquot of 850 µL of 20 µg/mL doxorubicin in PBS were added to the lower compartment. The plate was read continuously for 6 h in a fluorescence plate reader (PerkinElmer, Wallac II; Ex: 530 nm, Em: 570 nm).

For LOX modification assays, 140 µg (10 µg/mm^2^) of matrigel were mixed with 10 µg purified recombinant hmLOX or hLOXL2 in 50 µL PBS (±500 µM BAPN), the suspension was applied to transwell inserts (96-well MWD format, 14 mm^2^ membrane area, Costar, Cölbe, Germany) and incubated for 6 h at 37 °C. Afterward, the suspension was dried overnight and subjected to the assay described above (100 µL PBS in upper chamber, 300 µL 20 µg/mL doxorubicin in PBS in lower chamber).

### EC50 assay (FAK inhibitor PF-573228)

Cells were seeded into 96-well dishes at 10^3^ cells/well (E0771: 2.5 × 10^3^ cells/well) in 100 µL full media and incubated at 37 °C, 5% CO_2_, 2% O_2_. After 24 h, 100 µL of DMEM containing twice the indicated concentration of PF-573228 were added to each well without prior removal of medium. Each concentration was tested in a sixfold replicate. Cells were incubated with the therapeutics for 72 h before media was removed and cells were stored at −80 °C until further quantification using the CyQuant assay kit (Lifetechnologies, Carlsbad, CA) according to the manufacture’s instructions.

### Influence of substrate stiffness on cell proliferation

CytoSoft 6-well plates (Advanced Biomatrix, San Diego, CA) in six different degrees of stiffness (elastic modulus 0.5 to 64 kPa) were coated for 1 h with collagen I (Purecol, Advanced Biomatrix) in PBS at 100 µg/mL according to the manufacture's recommendations. Tumor cells were seeded at 3.0 × 10^4^ cells/well in 2 mL standard growth media (DMEM, 10% FBS). After 48 h, seeding efficacy was evaluated using a standard resazurin assay to quantify viable cells on the substrate: incubation with 25 µg/mL resazurin in 2 mL medium for 2 h at 37 °C and quantification of the fluorescent signal of generated resorufin in a plate reader (Ex: 520 nm, Em: 586 nm). The procedure was repeated 48–96 h later depending on the apparent growth rate of the particular cell line. Doubling time was calculated from the results obtained at the two time points and normalized to the growth rate in standard plastic 6-well cell culture plate (Corning).

### RNA isolation

RNA was isolated from cells using the RNeasy Kit (Qiagen, Hilden, Germany) according to the manufacturer’s recommendations.

RNA was isolated from fresh tumor samples using the Trizol reagent (Life Technologies, Darmstadt, Germany) according to the manufacturer’s recommendation.

### mRNA quantification

mRNA expression levels were quantified using the GeXP-System (BeckmanCoulter, Krefeld, Germany). Protocols for reverse transcription, amplification, labeling, gel electrophoresis, and quantification were used as recommended by the manufacturer. RNA levels were normalized to levels of housekeeping genes β-2-microglobulin (B2M) and ribosomal protein S29 (RPS29) [54]. Analysis was done with three technical replicates per biological sample. Mean values of technical replicates were used for statistical analysis.

### Statistical analysis

All statistical analysis was done using the Prism5 Software (GraphPad, LaJolla, CA). Differences between two groups were analyzed using an unpaired, two-tailed Student’s *t* test. In parallel, the samples were tested for significant variation of variance, and if necessary a Welch correction was included in the statistical analysis. For statistical analysis of metastatic incidence and size of metastases between groups, the Mann–Whitney test was used, as a Gaussian distribution could not be assumed. All statistical tests were performed between sets of individual biological replicates.

## Electronic supplementary material

Supplemental Data
