# Supplementary material for: LOX-catalyzed collagen stabilization is a proximal cause for intrinsic resistance to chemotherapy
Source: Oncogene. 2018 May 21;37(36):4921–40. doi: 10.1038/s41388-018-0320-2 (PMC6127085; doi:10.1038/s41388-018-0320-2)
Supplement: Supplementary file 1 — Supplemental Data [file 41388_2018_320_MOESM1_ESM.pdf]

LOX-catalyzed collagen stabilization is a proximal cause for intrinsic resistance to chemotherapy

Leonie Rossow[1#], Simona Veitl[1#], Sandra Vorlová[2#], Jacqueline K. Wax[1], Anja E. Kuhn[1], Verena Maltzahn[1], Berin Upcin[1,3], Franziska Karl[1,2], Helene Hoffmann[1,2], Sabine Gätzner[4], Matthias Kallius[1,6], Rajender Nandigama[1], Daniela Scheld[5], Ster Irmak[3], Sabine Herterich[5], Alma Zernecke[2], Süleyman Ergün[1], Erik Henke[1,6*]

# - Supplemental Data -

[1] Institute of Anatomy and Cell Biology II, Universität Würzburg, Koellikerstrasse 6, Würzburg 97070, Germany

[2] Institute of Experimental Biomedicine, Universitätsklinikum Würzburg, Josef-Schneider-Strasse 2/D16, 97082 Würzburg, Germany

[3] School of Health Sciences, Bilgi University Hacıahmet Mahallesi Pir Hüsamettin Sokak No:20 34440 Beyoğlu İstanbul, Turkey

[4] Institute of Tissue Engineering, Universität Würzburg, Roentgenring 11, Würzburg 97070, Germany

[5] Zentrallabor, Universitätsklinikum Würzburg, Josef-Schneider-Strasse 2, 97082 Würzburg, Germany

[6] Graduate School of Life Science, Universität Würzburg, Josef-Schneider-Strasse 2, 97082 Würzburg, Germany

# These authors contributed equally to this work

* To whom correspondence should be addressed:

Erik Henke, PhD

Institute for Anatomy and Cell Biology II, Universität Würzburg.

Koellikerstrasse 6

97070 Würzburg, Germany

Email: erik.henke@uni-wuerzburg.de

Tel: +49-(0)931-3183270, Fax: +49-(0)931-329363

Supplemental Table 1: Microarray Gene Expression Sets with Follow-Up

GEO Accession ID	Tissue	Treatment	# Samples Total/Resistant/Sensitive	Ref
**GSE25066**	BCA	Taxane/Antracycline	488/389/99	(3, 4)
**GDS4393**	CRC	FOLFOX	53[a)]/27/26	(5)
**GSE43502**	TN BCA	Taxane/Antracycline	25/16/9	(6)
**GSE20271**	BCA	Paclitaxel, 5-fluororacil, doxorubicin, cyclophosphamide	178/152/26	(7)
**GDS3297**	OVCa	Farmorubicin, Carboplatin, cyclophosphamid	54/34/20	(8)
**GDS3721**	BCA	Paclitaxel/Radiation	28/20/8	

[a)] Dataset contains paired primary and metastatic lesions. Analysis was performed on data from primary lesions.
BCA: Breast cancer, OVCa: Serous ovarian carcinoma CRC: Colorectal cancer, TN: Triple negative
FOLFOX: Leucovorin, 5-fluororacil, Oxaliplatin

Supplemental Table 2: ECM-related genes differentially expressed in resistant and sensitive tumors – Structural Proteins

Symbol	Name	GSE25066		GSE43502		GDS3721		GSE20271		GDS3297		GDS4393	
		Fold-Change	P-Value	Fold-Change	P-Value	Fold-Change	P-Value	Fold-Change	P-Value	Fold-Change	P-Value	Fold-Change	P-Value
**Collagens**													
COL1A1	collagen, type I, alpha 1	1.74	0.0011	1.22	0.0396					1.93	0.0486		
COL1A2	collagen, type I, alpha 2	1.79	0.0010			2.82	0.0015						
COL2A1	collagen, type II, alpha 1			1.34	0.0281	0.061	0.0373					1.3	0.0502
COL3A1	collagen, type III, alpha 1	1.88	0.0004			5.13	0.0009						
COL4A2	collagen, type IV, alpha 2	0.7	0.0018							1.32	0.017048		
COL4A3	collagen, type IV, alpha 3	1.28	0.0326	2.04	0.0211	14.08	0.0211					1.2	0.0486
COL4A5	collagen, type IV, alpha 5	1.48	0.0001			4.55	0.0196						
COL4A6	collagen, type IV, alpha 6	1.2	0.0172			16.14	0.0029						
COL5A1	collagen, type V, alpha 1	1.56	0.0041										
COL5A2	collagen, type V, alpha 2	1.49	0.0135			2.51	0.0198			1.40	0.0355		
COL5A3	collagen, type V, alpha 3					0.45	0.0449						
COL6A1	collagen, type VI, alpha 1			1.46	0.0019								
COL6A3	collagen, type VI, alpha 3	1.72	0.0003										
COL7A1	collagen, type VII, alpha 1			1.42	0.0349			0.90	0.0009				
COL8A1	collagen, type VIII, alpha 1					0.18	0.0360						
COL9A1	collagen, type IX, alpha 1	0.85	0.0381									1.43	0.0299
COL9A3	collagen, type IX, alpha 3	0.52	0.0001			0.23	0.0215						
COL10A1	collagen, type X, alpha 1	1.93	0.0000							1.33	0.0457		
COL11A1	collagen, type XI, alpha 1	1.66	0.0078										
COL12A1	collagen, type XII, alpha 1					13.51	0.0040					0.36	0.0393
COL14A1	collagen, type XIV, alpha 1									0.58	0.0318		
Col17A1	collagen, type XVII, alpha 1									1.24	0.0351		
COL19A1	collagen, type XIX, alpha 1	0.83	0.0247									4.72	0.0401
COL20A1	collagen, type XX, alpha 1			1.37	0.0012	6.37	0.0470						
COL21A1	collagen, type XXI, alpha 1									1.11	0.0208		
**Collagen Synthesis**													
BMP1	bone morphogenetic protein 1					0.18	0.0135			0.76	0.0417		

COLGALT1	collagen beta(1-O)galactosyltransferase 1	0.89	0.0042	1.47	0.0364								
ERO1L	ERO1-like (S. cerevisiae)			1.13	0.0259	25.14	0.0000						
LOX	lysyl oxidase			1.3	0.0485								
LOXL1	lysyl oxidase-like 1	1.87	0.0001										
LOXL2	lysyl oxidase-like 2							1.12	0.0451				
P4HB	prolyl 4-hydroxylase, beta polypeptide	0.86	0.0132									1.12	0.0472
PCOLCE	procollagen C-endopeptidase enhancer	1.34	0.0186										
PLOD1	procollagen-lysine, 2-oxoglutarate 5-dioxygenase 1	0.78	0.0000										
PLOD2	procollagen-lysine, 2-oxoglutarate 5-dioxygenase 2	0.66	0.0001									1.23	0.0399
PLOD3	procollagen-lysine, 2-oxoglutarate 5-dioxygenase 3					0.3	0.0222					0.87	0.0100
TLL1	tolloid-like 1												
TLL2	tolloid-like 2	1.28	0.0173									0.61	0.0155
**Laminins**													
LAMA1	laminin, alpha 1												
LAMA2	laminin, alpha 2			1.1	0.0323							2.03	0.0100
LAMA3	laminin, alpha 3	1.66	0.0003			3.66	0.0350			1.89	0.0441		
LAMA4	laminin, alpha 4	1.18	0.0435									1.35	0.0107
LAMA5	laminin, alpha 5	1.27	0.0001					1.19	0.0004				
LAMB1	laminin, beta 1												
LAMB2	laminin, beta 2	1.34	0.0000					1.19	0.0122				
LAMC1	laminin, gamma 1									1.29	0.0365		
**Proteoglycans**													
BCAN	brevican			2.13	0.0447								
BGN	biglycan	1.29	0.0153										
DCN	decorin	1.48	0.0049										
FMOD	fibromodulin	1.33	0.0001										
GPC1	glypican 1	1.42	0.0000					1.09	0.0276				
GPC2	glypican 2			0.71	0.0023								
GPC4	glypican 4							1.16	0.0205				
HSPG2	heparan sulfate proteoglycan 2			1.39	0.0233								
IMPG2	interphotoreceptor matrix proteoglycan 2	1.41	0.0006										
LEPREL1	leprecan-like 1												
LEPREL2	leprecan-like 2	1.23	0.0152										
LUM	lumican	1.77	0.0066										
PODNL1	podocan-like 1							1.27	0.0122				
SDC2	syndecan 2												
SDC3	syndecan 3											0.85	0.0470
SDC4	syndecan 4	1.23	0.0015										
SPOCK1	sparc/osteonectin, cwcv and kazal-like domains proteoglycan (testican) 1	1.42	0.0002										
SPOCK2	sparc/osteonectin, cwcv and kazal-like domains proteoglycan (testican) 2							0.70	0.0018				
VCAN	versican	1.35	0.0394										

**Tenascins**													
TNN	tenascin N					3.03	0.0267						
TNC	tenascin C					0.22	0.0302						
TNR	tenascin R	1.1	0.0584			3.02	0.0282			1.32	0.0419		
TNXB	tenascin XB					9.31	0.0433						
**ECM**													
AMELY	amelogenin, Y-linked			1.93	0.0565								
ASPN	asporin	1.95	0.0002										
DMD	dystrophin	0.73	0.0152					0.74	0.0187			1.32	0.0499
DPT	dermatopontin	1.3	0.0468					1.23	0.0319				
ECM1	extracellular matrix protein 1	1.65	< 1e-07										
ECM2	extracellular matrix protein 2, female organ and adipocyte specific												
EFEMP1	EGF containing fibulin-like extracellular matrix protein 1												
EFEMP2	EGF containing fibulin-like extracellular matrix protein 2	1.25	0.0001										
FBLN1	fibulin 1	0.74	0.0297										
FBLN2	fibulin 2	0.63	0.0047			0.28	0.0132						
FBLN5	fibulin 5	0.72	0.0041					0.83	0.0062				
FN1	fibronectin 1					35.14	0.0133						
FREM3	FRAS1 related extracellular matrix 3											3.92	0.0150
HMCN2	hemicentin 2			1.22	0.0086								
MGP	matrix Gla protein												
NID1	nidogen 1									0.71	0.0036		
PRELP	proline/arginine-rich end leucine-rich repeat protein			2.09	0.0121								
SPON1	spondin 1, extracellular matrix protein												
**Elastin**													
ELN	elastin	0.58	0.0000			0.25	0.0247						
FBN1	fibrillin 1	1.39	0.0247										
FBN2	fibrillin 2	1.23	0.0083										
FBN3	fibrillin 3					0.19	0.0237						
**Hyaluronan**													
HAPLN1	hyaluronan and proteoglycan link protein 1					0.31	0.0395						
HAPLN2	hyaluronan and proteoglycan link protein 2												
HAPLN4	hyaluronan and proteoglycan link protein 4			1.47	0.0482								
HAS1	hyaluronan synthase 1			2.06	0.0184							0.23	0.0248
HAS2	hyaluronan synthase 2					0.084	0.0398						
HAS3	hyaluronan synthase 3												
**Amyloid Beta**													
APBB2	amyloid beta (A4) precursor protein-binding, family B, member 2	1.59	0.0006			11.03	0.0316	1.32	0.0083				
APLP2	amyloid beta (A4)									2.63	0.00015		

	precursor-like protein												
APLP2	amyloid beta (A4) precursor-like protein 2	1.16	0.0047										
APP	amyloid beta (A4) precursor protein					0.3	0.0177						
PSEN1	presenilin 1	1.1	0.0057							1.34	0.00052	1.22	0.0495

Supplemental Table 3: ECM-related genes differentially expressed in resistant and sensitive tumors – Proteolytic Proteins and Inhibitors

Symbol	Name	GSE25066		GSE43502		GDS3721		GSE20271		GDS3297		GDS4393	
		Fold-Change	P-Value	Fold-Change	P-Value	Fold-Change	P-Value	Fold-Change	P-Value	Fold-Change	P-Value	Fold-Change	P-Value
**ADAMs**													
ADAM8	ADAM metallopeptidase domain 8			0.81	0.0444								
ADAM9	ADAM metallopeptidase domain 9									0.64	0.0162		
ADAM10	ADAM metallopeptidase domain 10	1.11	0.0161										
ADAM11	ADAM metallopeptidase domain 11												
ADAM15	ADAM metallopeptidase domain 15	0.75	0.0268										
ADAM17	ADAM metallopeptidase domain 17	0.85	0.0022							1.73	0.0002		
ADAMTS2	ADAM metallopeptidase with thrombospondin type 1 motif, 2							0.87	0.0433895				
ADAMTS6	ADAM metallopeptidase with thrombospondin type 1 motif, 6	1.15	0.0353										
ADAMTS7	ADAM metallopeptidase with thrombospondin type 1 motif, 7			2.46	0.0544								
ADAMTS8	ADAM metallopeptidase with thrombospondin type 1 motif, 8											0.73	0.0476
ADAMTS9	ADAM metallopeptidase with thrombospondin type 1 motif, 9			0.8	0.0470								
ADAMTS13	ADAM metallopeptidase with thrombospondin type 1 motif, 13					11.61	0.0148						
ADAMTS15	ADAM metallopeptidase with thrombospondin type 1 motif, 15			1.98	0.0082							0.74	0.0071
ADAMTS19	ADAM metallopeptidase with											1.29	0.0379

	thrombospondin type 1 motif, 19												
ADAMTSL1	ADAMTS-like 1					7.1	0.0209					1.39	0.0003
ADAMTSL2	ADAMTS-like 2									0.82	0.0174		
ADAMTSL3	ADAMTS-like 3					0.23	0.0280						
ADAMTSL4	ADAMTS-like 4					7.44	0.0405						
**MMPs**													
MMP2	matrix metallopeptidase 2 (gelatinase A, 72kDa gelatinase, 72kDa type IV collagenase)	1.34	0.0066										
MMP7	matrix metallopeptidase 7 (matrilysin, uterine)	0.58	0.0003										
MMP8	matrix metallopeptidase 8 (neutrophil collagenase)											0.81	0.0110
MMP10	matrix metallopeptidase 10 (stromelysin 2)	1.37	0.0173							1.33	0.0148		
MMP11	matrix metallopeptidase 11 (stromelysin 3)	1.55	0.0002					0.92	0.0209695			0.81	0.0187
MMP12	matrix metallopeptidase 12 (macrophage elastase)	0.68	0.0030										
MMP16	matrix metallopeptidase 16 (membrane-inserted)												
MMP17	matrix metallopeptidase 17 (membrane-inserted)	1.24	0.0187	1.61	0.0021								
MMP20	matrix metallopeptidase 20												
MMP24	matrix metallopeptidase 24 (membrane-inserted)							1.2	0.0033569				
MMP25	matrix metallopeptidase 25											0.82	0.0010
MMP28	matrix metallopeptidase 28			1.54	0.0096			2.26	0.0344401				
**Kallikrein Proteases**													
KLK2	kallikrein-related peptidase 2							1.14	0.0335068				
KLK4	kallikrein-related peptidase 4											0.8	0.0345
KLK7	kallikrein-related peptidase 7	0.61	0.0001	0.11	0.0346					1.71	0.0292		
**Cathepsins**													
CTSB	cathepsin B					13.96	0.0001	0.75	0.0492617				
CTSD	cathepsin D					0.51	0.0391						
CTSG	cathepsin G									0.84	0.0058		
CTSK	cathepsin K	1.32	0.0036										
CTSS	cathepsin S							0.75	0.0269438				

CTSV	cathepsin V	0.6	< 1e-07										
**Misc Proteases**													
CTRB2	chymotrypsinogen B2									1.41	0.0235		
FURIN	furin (paired basic amino acid cleaving enzyme)			1.16	0.0388								
HTRA1	HtrA serine peptidase 1	1.39	0.0004										
PRSS2	protease, serine, 2 (trypsin 2)							1.09	0.0372451				
TMPRSS6	transmembrane protease, serine 6	1.22	0.0000										
TPSAB1	tryptase alpha/beta 1	1.86	0.0000			4.6	0.0166						
PRTN3	proteinase 3												
**Serpines**													
SERPINA1	serpin peptidase inhibitor, clade A (alpha-1 antiproteinase, antitrypsin), member 1	1.52	0.0026	1.54	0.0362	6.3	0.0076						
SERPINB5	serpin peptidase inhibitor, clade B (ovalbumin), member 5	0.61	0.0065										
SERPINE2	serpin peptidase inhibitor, clade E (nexin, plasminogen activator inhibitor type 1), member 2	0.59	0.0000							0.59	0.0162		
SERPINH1	serpin peptidase inhibitor, clade H (heat shock protein 47), member 1, (collagen binding protein 1)	0.54	0.0007							1.39	0.0158		
**TIMPs**													
TIMP1	TIMP metallopeptidase inhibitor 1	1.38	0.0001										
TIMP3	TIMP metallopeptidase inhibitor 3	1.84	0.0000			26.71	0.0023						
**Kazal Domain Protease Inhibitors**													
AGRN	agrin					5.23	0.0393						
IGFBP7	insulin-like growth factor binding protein 7	1.52	0.0399										
KAZALD1	Kazal-type serine peptidase inhibitor domain 1	1.31	0.0146							1.56	0.0167		
RECK	reversion-inducing-cysteine-rich protein with kazal motifs					5.68	0.0419						
SMOC1	SPARC related modular calcium binding 1					0.19	0.0383						

SMOC2	SPARC related modular calcium binding 2												
SPARC	secreted protein, acidic, cysteine-rich (osteonectin)	1.53	0.0023										
SPARCL1	SPARC-like 1 (hevin)												
**Other Protease Inhibitors**													
AGT	angiotensinogen (serpin peptidase inhibitor, clade A, member 8)	1.34	0.0001			0.24	0.0351						
CST3	cystatin C	1.81	< 1e-07							1.68	0.0001		
PI3	peptidase inhibitor 3, skin-derived			2	0.0401							0.32	0.0344
SPINT1	serine peptidase inhibitor, Kunitz type 1			2.25	0.0384								
SLPI	secretory leukocyte peptidase inhibitor	0.64	0.0001										

Supplemental Table 4: ECM-related genes differentially expressed in resistant and sensitive tumors – Miscellaneous

Symbol	Name	GSE25066 Fold-Change	GSE25066 P-Value	GSE43502 Fold-Change	GSE43502 P-Value	GDS3721 Fold-Change	GDS3721 P-Value	GSE20271 Fold-Change	GSE20271 P-Value	GDS43297 Fold-Change	GDS43297 P-Value	GDS4393 Fold-Change	GDS4393 P-Value
**Adhesion**													
CD151	CD151 molecule (Raph blood group)							1.23	0.0236	1.38	0.0191		
CD248	CD248 molecule, endosialin												
DST	dystonin					0.24	0.0360						
F11R	F11 receptor	0.87	0.0031					1.12	0.0385				
ICAM1	intercellular adhesion molecule 1	0.85	0.0091	1.28	0.0402	0.17	0.0400	0.81	0.0158				
ICAM2	intercellular adhesion molecule 2							0.88	0.0067				
ICAM3	intercellular adhesion molecule 3	1.19	0.0216							1.77	0.0144		
JAM2	junctional adhesion molecule 2												
JAM3	junctional adhesion molecule 3	1.25	0.0018	1.22	0.0424								
MADCAM1	mucosal vascular addressin cell adhesion molecule 1	1.13	0.0166										
NCAM1	neural cell adhesion molecule 1					0.14	0.0413						
NRXN1	neurexin 1	2.47	0.0320										
VCAM1	vascular cell adhesion molecule 1	0.83	0.0425					0.62	0.0014				
**Integrins**													
IBSP	integrin-binding sialoprotein					4.5	0.0472						
ITGA1	ntegrin subunit alpha 1 (VLA1 receptor; CD49a)									0.73	0.0389		
ITGA10	integrin alpha 10									1.21	0.0124		
ITGA11	integrin, alpha 11					0.2	0.0113						
ITGA2	integrin, alpha 2 (CD49B, alpha 2 subunit of VLA-2 receptor)	1.13	0.0467										
ITGA3	integrin, alpha 3 (antigen CD49C, alpha 3 subunit of VLA-3 receptor)	1.34	0.0167										
ITGA4	integrin, alpha 4 (antigen CD49D, alpha 4 subunit of VLA-4 receptor)					13.53	0.0062	0.78	0.0029				
ITGA5	integrin, alpha 5 (fibronectin receptor, alpha polypeptide)												
ITGA6	integrin, alpha 6	0.51	0.0000										
ITGA7	integrin, alpha 7												
ITGA8	integrin, alpha 8	1.22	0.0084	1.8	0.0220								
ITGA9	integrin, alpha 9											1.78	0.0019
ITGAE	Integrin, alpha E (CD103, endothelial associated)									2.4	0.013		

ITGAL	integrin, alpha L (antigen CD11A (p180), lymphocyte function-associated antigen 1; alpha polypeptide)												
ITGAM	integrin, alpha M (complement component 3 receptor 3 subunit)	1.11	0.0433										
ITGAV	integrin, alpha V	1.16	0.0323										
ITGB1	integrin, beta 1 (fibronectin receptor, beta polypeptide, antigen CD29 includes MDF2, MSK12)	0.71	0.0007										
ITGB2	integrin, beta 2 (complement component 3 receptor 3 and 4 subunit)					0.23	0.0174	0.65	0.0070			1.32	0.0260
ITGB3	integrin, beta 3 (platelet glycoprotein IIIa, antigen CD61)					7.16	0.0491	1.10	0.0272	0.38	0.0007		
ITGB4	integrin, beta 4	0.79	0.0286	1.77	0.0021					0.59	0.0017		
ITGB5	integrin, beta 5	1.6	0.0000										
ITGB7	integrin, beta 7					0.19	0.0203						
ITGB8	integrin, beta 8	0.87	0.0499			0.21	0.0450						
POSTN	periostin, osteoblast specific factor	1.75	0.0003										
**Galectins**													
LGALS1	lectin, galactoside-binding, soluble, 1			1.19	0.0385								
LGALS3	lectin, galactoside-binding, soluble, 3			1.11	0.0302	8.67	0.0222						
LGALS3BP	lectin, galactoside-binding, soluble, 3 binding protein												
**TGF-beta Pathway**													
BMP2	bone morphogenetic protein 2					0.25	0.0107	0.86	0.0233				
BMP7	bone morphogenetic protein 7												
CER1	cerberus 1, DAN family BMP antagonist					3.64	0.0353						
LTBP1	latent transforming growth factor beta binding protein 1	0.61	0.0000			4.14	0.0492						
LTBP2	latent transforming growth factor beta binding protein 2	1.19	0.0273										
LTBP4	latent transforming growth factor beta binding protein 4			1.39	0.0110								
NBL1	neuroblastoma 1, DAN family BMP antagonist	1.42	0.0110										
SMAD3	SMAD family member 3	1.23	0.0001	0.86	0.0084			1.15	0.0088	1.23	0.0419		
TGFB1	transforming growth factor, beta 1	1.19	0.0030										
TGFB1I1	transforming growth factor beta 1 induced transcript 1	1.28	0.0011										
TGFB2	transforming growth factor, beta 2											0.84	0.0369
TGFB3	transforming growth factor, beta 3	1.44	< 1e-07			5.03	0.0376						
TGFBI	transforming growth factor, beta-induced, 68kDa					0.33	0.0288						
**Thrombospondin**													

CD47	CD47 molecule	0.73	0.0119	1.07	0.0438	11.86	0.0149			1.42	0.0269		
COMP	cartilage oligomeric matrix protein	2.24	0.0000										
THBS1	thrombospondin 1	1.55	< 1e-07	1.25	0.0252								
THBS4	thrombospondin 4	1.22	0.0044										
**Transcription Factors**													
ELF3	E74-like factor 3 (ets domain transcription factor, epithelial-specific)					2.07	0.0360	1.15	0.0483				
ETS1	v-ets avian erythroblastosis virus E26 oncogene homolog1			1.22	0.0231								
FOXF1	forkhead box F1												
TCF15	transcription factor 15 (basic helix-loop-helix)									1.58	0.0029		
SOX9	SRY (sex determining region Y)-box 9	0.76	0.0024										
**VW-Domain**													
MATN3	matrilin 3	1.91	0.0000					1.79	0.0071				
VWA1	von Willebrand factor A domain containing 1					0.25	0.0387						
VWF	von Willebrand factor												
**Wnt-Pathway**													
SFRP1	secreted frizzled-related protein 1	0.36	< 1e-07					0.68	0.0346				
WNT1	wingless-type MMTV integration site family, member 1												
WNT10A	wingless-type MMTV integration site family, member 10A					0.26	0.0111						
WNT11	wingless-type MMTV integration site family, member 11					0.3	0.0284						
WNT2	wingless-type MMTV integration site family member 2												
WNT2B	wingless-type MMTV integration site family, member 2B	0.83	0.0271	4	0.0158								
WNT4	wingless-type MMTV integration site family, member 4	1.5	0.0030										
WNT5A	wingless-type MMTV integration site family, member 5A	1.3	0.0398			9.11	0.0222						
WNT5B	wingless-type MMTV integration site family, member 5B			1.33	0.0423								
WNT6	wingless-type MMTV integration site family, member 6	0.76	0.0107	1.27	0.0427	0.25	0.0241						
WNT7B	wingless-type MMTV integration site family, member 7B												
WNT8A	wingless-type MMTV integration site family, member 8A												
WNT8B	wingless-type MMTV integration site family, member 8B												
**Mucins**													
MFGE8	milk fat globule-EGF factor 8 protein	0.58	0.0000										
MUC3A	mucin 3A, cell surface	1.1	0.027									0.6	0.0405

	associated		1									4	
MUC4	mucin 4, cell surface associated			2.02	0.0509			1.11	0.0271				
MUC5AC	mucin 5AC, oligomeric mucus/gel-forming	1.23	0.0434					1.16	0.0266	0.68	0.0174	0.85	0.0493
SFTPA2	surfactant protein A2												
	surfactant proteinD									0.37	0.0037		

Miscancealous

PLSCR1	phospholipid scramblase 1	0.68	0.0000					0.79	0.0392	1.53	0.0198		
A2M	alpha-2-macroglobulin												
ABI3BP	ABI family, member 3 (NESH) binding protein												
ACTN1	actinin, alpha 1			1.23	0.0277								
AEBP1	AE binding protein 1	1.53	0.0003										
ANGPTL4	angiopoietin-like 4												
ANXA2	annexin A2	1.11	0.0401	1.53	0.0030								
ALPL	alkaline phosphatase												
APOE	apolipoprotein E			1.36	0.0085			0.58	0.0088			2.92	0.0327
APOH	apolipoprotein H												
ATP7A	ATPase, Cu++ transporting, alpha polypeptide	1.15	0.0008										
BCL3	B-cell CLL/lymphoma 3	1.28	0.0003										
BSG	basigin (Ok blood group)												
C6orf15	chromosome 6 open reading frame 15												
CALR	calreticulin	0.77	0.0074	1.16	0.0029	3.38	0.0311					0.89	0.0298
CASK	calcium/calmodulin-dependent serine protein kinase (MAGUK family)	0.78	0.0487			0.17	0.0245						
CASP3	caspase 3, apoptosis-related cysteine peptidase	0.91	0.0186										
CCBE1	collagen and calcium binding EGF domains 1			2.02	0.0006								
CCDC80	coiled-coil domain containing 80												
CD4	CD4 molecule							0.90	0.0200				
CD44	CD44 molecule (Indian blood group)							1.11	0.0176				
CDON	cell adhesion associated, oncogene regulated			0.78	0.0546								
CFP	complement factor properdin					0.18	0.0454						
CHAD	chondroadherin	2	0.0000			15.32	0.0056						
CHI3L1	chitinase 3-like 1 (cartilage glycoprotein-39)	0.57	0.0035										
CIB1	calcium and integrin binding 1 (calmyrin)	1.28	0.0000										
CILP	cartilage intermediate layer protein, nucleotide pyrophosphohydrolase	1.28	0.0023										
CLU	clusterin	0.83	0.0211							0.38	0.0003		
CMA1	chymase 1									1.21	0.035		
COCH	cochlin	0.57	0.0000	0.7	0.0341	0.11	0.0422						
CRIP2	cysteine-rich protein 2	1.3	0.0075										

CRISP3	cysteine-rich secretory protein 3											4.82	0.0436
CRISPLD2	cysteine-rich secretory protein LCCL domain containing 2			1.31	0.0545								
CRTAP	cartilage associated protein	1.14	0.0011			0.21	0.0238						
CSGALNACT1	chondroitin sulfate N-acetylgalactosaminyltransferase 1												
CTGF	connective tissue growth factor												
CTHRC1	collagen triple helix repeat containing 1					8.53	0.0268						
CYP1B1	cytochrome P450, family 1, subfamily B, polypeptide 1	0.79	0.0390			0.28	0.0436	0.60	0.0103				
DDR2	discoidin domain receptor tyrosine kinase 2	0.79	0.0111										
EMID1	EMI domain containing 1			0.59	0.0146			1.11	0.0345				
FAP	fibroblast activation protein, alpha	1.49	0.0020										
FER	fer (fps/fes related) tyrosine kinase					10.45	0.0399					1.36	0.0266
FGFR2	fibroblast growth factor receptor 2			0.59	0.0398	0.21	0.0426			1.94	0.0418		
FGFR4	fibroblast growth factor receptor 4			1.45	0.0260								
FLOT1	flotillin 1	1.17	0.0017										
FLRT3	fibronectin leucine rich transmembrane protein 3	1.36	0.0003					1.19	0.0218				
GLG1	golgi glycoprotein 1	0.91	0.0375										
GPLD1	glycosylphosphatidylinositol specific phospholipase D1												
GPM6B	glycoprotein M6B	0.35	< 1e-07					0.75	0.0058				
HNRNPM	heterogeneous nuclear ribonucleoprotein M	0.89	0.0013										
HSD17B12	hydroxysteroid (17-beta) dehydrogenase 12	0.87	0.0087										
HSP90B1	heat shock protein 90kDa beta (Grp94), member 1	0.9	0.0454										
IHH	indian hedgehog												
KAL1	Kallmann syndrome 1 sequence	1.48	< 1e-07										
KIF9	kinesin family member 9					19.74	0.0094						
KLKB1	kallikrein B, plasma (Fletcher factor) 1											1.3	0.0265
KRT1	keratin 1												
LECT1	leukocyte cell derived chemotaxin 1												
LEFTY2	left-right determination factor 2			1.86	0.0271								
LPL	lipoprotein lipase	0.63	0.0142										
LRP4	low density lipoprotein receptor-related protein 4			0.54	0.0079								
LRP5	low density lipoprotein receptor-related protein 5					2.79	0.0310						
MFAP1	microfibrillar-associated protein 1	1.16	< 1e-07										
MFAP2	microfibrillar-associated protein 2	0.62	0.0003							1.81	0.0023		

MFAP3	microfibrillar-associated protein 3			1.14	0.0519					1.25	0.0321		
MFAP4	microfibrillar-associated protein 4												
MFAP5	microfibrillar associated protein 5												
MFI2	antigen p97 (melanoma associated) identified by monoclonal antibodies 133.2 and 96.5	0.74	0.0001									1.81	0.0344
MPZL3	myelin protein zero-like 3			0.7	0.0539								
NDNF	neuron-derived neurotrophic factor												
NDP	Norrie disease (pseudoglioma)	1.21	0.0107										
NF1	neurofibromin 1	1.12	0.0283	0.93	0.0354	9.64	0.0032						
NFKB2	nuclear factor of kappa light polypeptide gene enhancer in B-cells 2 (p49/p100)	0.91	0.0285							1.34	0.0200		
NOTCH1	notch 1	0.77	0.0001										
NOV	nephroblastoma overexpressed			1.54	0.0110	0.22	0.0392	0.83	0.0226				
NOX1	NADPH oxidase 1			1.33	0.0110								
NOXO1	NADPH oxidase organizer 1											0.66	0.0453
NPNT	nephronectin					10.11	0.0045						
NR2E1	nuclear receptor subfamily 2, group E, member 1											0.8	0.0081
NTN4	netrin 4											1.71	0.0068
OLFML2A	olfactomedin-like 2A												
OLFML2B	olfactomedin-like 2B												
PDGFB	platelet-derived growth factor beta polypeptide	1.16	0.0004			0.35	0.0295	1.08	0.0225				
PECAM1	platelet/endothelial cell adhesion molecule 1					5.51	0.0345	0.83	0.0238				
PKM	pyruvate kinase, muscle			1.16	0.0170								
PLEC	plectin	1.24	0.0223										
PLG	plasminogen							0.93	0.0094				
POMT1	protein-O-mannosyltransferase 1											1.27	0.0322
PRDX4	peroxiredoxin 4	0.73	< 1e-07										
PRKCA	protein kinase C, alpha					0.13	0.0036						
PTK2	protein tyrosine kinase 2												
PTPRS	protein tyrosine phosphatase, receptor type, S							1.09	0.0328				
PTPRZ1	protein tyrosine phosphatase, receptor-type, Z polypeptide 1	0.78	0.0088					0.87	0.0159				
RELN	reelin	0.81	0.0183	0.65	0.0128					0.66	0.0050		
RGCC	regulator of cell cycle	0.76	0.0114										
RXFP1	relaxin/insulin-like family peptide receptor 1			0.66	0.0214								
SERAC1	serine active site containing 1			1.49	0.0236								
SH3PXD2B	SH3 and PX domains 2B			1.16	0.0362								
SHH	sonic hedgehog												

SMARCA4	SWI/SNF related, matrix associated, actin dependent regulator of chromatin, subfamily a, member 4	0.8	0.0208	0.83	0.0184								
STATH	statherin					3.22	0.0196						
TGM4	transglutaminase 4			1.54	0.0305	37.86	0.0004						
TNFRSF11B	tumor necrosis factor receptor superfamily, member 11b							0.81	0.0063				
VEGFA	vascular endothelial growth factor A	0.61	2E-05										
EGFL6	EGF-like-domain, multiple 6									1.5	0.014		
ZG16	zymogen granule protein 16					4.32	0.0148						
ZP1	zona pellucida glycoprotein 1 (sperm receptor)			1.16	0.0300								
ZP2	zona pellucida glycoprotein 2 (sperm receptor)			2.48	0.0567								
ZP4	zona pellucida glycoprotein 4												
TECTA	tectorin alpha												

Supplemental Table 5: Collagen Synthesis Genelist

Symbol	EntrezID	Name	Function
COL1A1	1277	Collagen type 1 α1	
COL1A2	1278	Collagen type 1 α2	
COL2A1	1280	Collagen type 2 α1	
COL3A1	1281	Collagen type 3 a1	
COL4A1	1282	Collagen type 4 α1	
COL4A2	1284	Collagen type 4 α2	
COL4A3	1285	Collagen type 4 α3	
COL4A4	1286	Collagen type 4 α4	
COL4A5	1287	Collagen type 4 α5	
COL4A6	1288	Collagen type 4 α6	
COL5A1	1289	Collagen type 5 a1	
COL5A2	1290	Collagen type 5 a2	
COL5A3	50509	Collagen type 5 a3	
COL6A1	1291	Collagen type 6 a1	
COL6A2	1292	Collagen type 6 a2	
COL6A3	1293	Collagen type 6 a3	
COL7A1	1294	Collagen type 7 a1	
COL8A1	1295	Collagen type 8 a1	
COL8A2	1296	Collagen type 8 a2	
COL9A1	1297	Collagen type 9 a1	
COL9A2	1298	Collagen type 9 a2	
COL9A3	1299	Collagen type 9 a3	
COL10A1	1300	Collagen type 10 a1	
COL11A1	1301	Collagen type 11 a1	
COL11A2	1302	Collagen type 12 a1	
COL13A1	1305	Collagen type 13 a1	
COL14A1	7373	Collagen type 14 a1	
COL15A1	1306	Collagen type 15 a1	
COL16A1	1307	Collagen type 16 a1	
COL17A1	1308	Collagen type 17 a1	
COL18A1	80781	Collagen type 18 a1	
COL19A1	1310	Collagen type 19 a1	
COL21A1	81578	Collagen type 21 a1	
ASPH	444	Aspartyl/asparaginyl beta-hydroxylase	Calcium Binding
BMP1	649	Bone Morphogenic Protein-1	Co- and post-translational modification
CALR	811	Calreticulin	N-Glycosylation and Folding
COLGALT1	79709	Collagen β(1-O)galactosyltransferases	Galoctosylation
COLGALT2	23127	Collagen β(1-O)galactosyltransferases	Galoctosylation
CRTAP	10491	Cartilage-associated protein	Co- and post-translational modification
ERO1L	30001	ERO1-like protein alpha	Disulfide Bond Formation
FKBP10	60681	Peptidyl-prolyl cis-trans FKBP65	Peptidyl-prolyl isomerase
FKBP9	11328	Peptidyl-prolyl cis-trans FKBP9	Peptidyl-prolyl isomerase
GANAB	23193	glucosidase 2 alpha subunit	N-Glycosylation and Folding
LEPRE1	64175	Prolyl-3-Hydroxylase 1 (P3H1)	Co- and post-translational modification
LEPREL2	10536	Prolyl-3-Hydroxylase 3 (P3H3)	Co- and post-translational modification
LOX	4015	Lysyl Oxidase	Co- and post-translational modification
LOXL1	4016	Lysyl Oxidase Like1	Co- and post-translational modification
LOXL1-AS1	100287616	Lysyl Oxidase Like1 Antisense	Co- and post-translational modification
LOXL2	4017	Lysyl Oxidase Like2	Co- and post-translational modification
LOXL3	84695	Lysyl Oxidase Like3	Co- and post-translational modification
LOXL4	84171	Lysyl Oxidase Like4	Co- and post-translational modification
P4HA1	5033	Prolyl- 4-hydroxilase-α1	Co- and post-translational modification
P4HA2	8974	Prolyl- 4-hydroxilase-α2	Co- and post-translational modification
P4HA3	283208	Prolyl- 4-hydroxilase-3	Co- and post-translational modification
P4HB	5034	Proteindissulfid isomerase	Co- and post-translational modification
PCOLCE	5118	Procollagen C-endopeptidase enhancer 1	Co- and post-translational modification

PCOLCE2	26577	Procollagen C-endopeptidase enhancer 2	Co- and post-translational modification
PLOD1	5351	Procollagen-lysine,2-oxoglutarate 5-	Co- and post-translational modification
PLOD2	5352	Procollagen-lysine,2-oxoglutarate 5-	Co- and post-translational modification
PLOD3	8985	Procollagen-lysine,2-oxoglutarate 5-	Co- and post-translational modification
PPIB	5479	Peptidyl-prolyl cis-trans isomerase B	Peptidyl-prolyl isomerase B
PRKCSH	5589	Glucosidase-2 β subunit	N-Glycosylation and Folding
SERPINH1	871	Heat shock protein 47	Collagen-Specific Chaperone
UGGT1	56886	UDP-glucose glucosyltranferase 1	N-Glycosylation and Folding

Supplemental Table 6: Differentially expressed collagen synthesis genes.

		GSE25066			GSE43502			GDS4393			GSE20271			GDS3297		
	Agent[a)]	Res_{High} vs. Res_{Low}	Res_{High} vs. Sens.	Res_{Low} vs. Sens.	Res_{High} vs. Res_{Low}	Res_{High} vs. Sens.	Res_{Low} vs. Sens.	Res_{High} vs. Res_{Low}	Res_{High} vs. Sens.	Res_{Low} vs. Sens.	Res_{High} vs. Res_{Low}	Res_{High} vs. Sens.	Res_{Low} vs. Sens.	Res_{High} vs. Res_{Low}	Res_{High} vs. Sens.	Res_{Low} vs. Sens.
**Procollagen C proteinase**																
BMP1	Sagramostin<br>UK 383367	x	x					x	x					x		x
PCOLCE		x	x								x	x	x	x		x
PCOLCE2			x	x				x								
**Collagen β(1-O)galactosyltransferases**																
COLGALT1	-	x		x	x			x								
COLGALT2		x			x	x										
**Prolyl-3-Hydroxylases**																
LEPRE1	-	x	x								x	x	x	x		
LEPREL2	-	x	x					x								x
**Lysyl oxidases**																
LOX	3-Amino propionitrile(9)	x	x	x	x	x		x	x	x	x	x	x	x	x	
LOXL1		x	x					x	x		x	x		x	x	
LOXL2		x	x	x	x	x		x	x		x	x	x	x		x
**Prolyl-4-hydroxylases**																
P4HA1	Ascorbic Acid<br>1,4-DPCA(10)						x		x	x				x		
P4HA2		x	x					x	x	x				x		x
P4HB		x														
**Procollagen-lysine,2-oxoglutarate 5-dioxygenases**																
PLOD1	Ascorbic Acid	x		x												
PLOD2		x		x					x	x				x		
PLOD3		x														
**Miscellaneous**																
CALR	Melatonin														x	x
CRTAP		x	x	x											x	
ERO1L	Flavin adenine dinucleotide<br>Ethyl-pyrrolidine-2,5-Dione	x							x	x						
GANAB	Miglitol			x	x											
FKBP9	GPI-1046<br>GPI1485<br>L-709587<br>Sirolimus<br>Pimecrolimus	x			x	x		x								
PRKCSH	Miglitol														x	
SERPINH1	SR-123781<br>Merck385874(11)<br>Xantholipin	x		x			x		x	x	x	x	x	x	x	
UGGT1	DL-*threo*-PDMP(12)			x	x		x									

a) according to DrugBank entry, or specific reference.

x: Genes significantly ($P < 0.05$) expressed.
Genes significantly higher expreessed in Res_{High} versus both Res_{Low} and sensitive Tumors, but not in ResLow vs. sensitive tumors are marked in green.

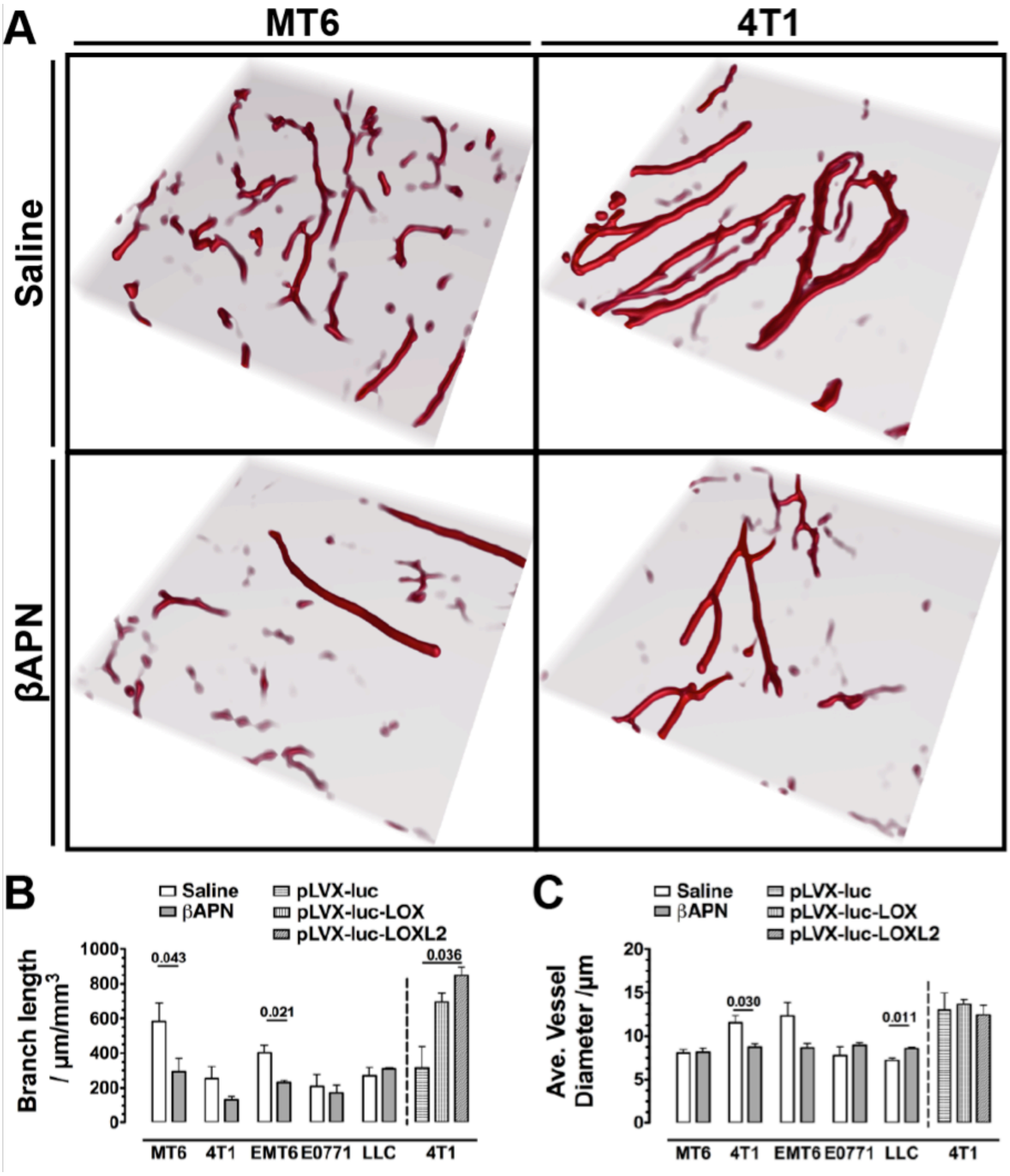

Supplemental Figure S1: Vascular changes after βAPN-treatment or LOX/LOX2 OE:

(A) 3D angiographies of MT6 and 4T1 tumor sections before and after βAPN treatment. Lysyl oxidase inhibition reduced perfused vessel density and resulted in a clearer, less branched vasculature. Confocal images of vessels stained *in vivo* with Isolectin GS-B4. Stack size: 637 x 637 x 45 µm.

(B) Total 3D branch length/volume in tumors after βAPN treatment or LOX/LOXL2 OE (n = 4).

(C) Average vessel diameter in tumors after βAPN treatment or LOX/LOXL2 OE (n = 4).

Error bars: ±SEM

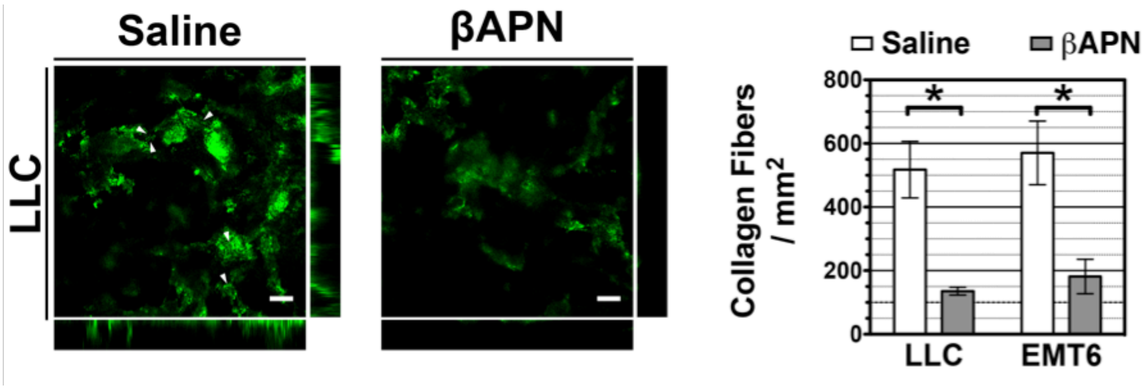

Figure S2 Reduction of collagen crosslinking in ECM extracts from experimental tumors after lysyl oxidase inhibition: ECM from βAPN-treated and control LLC and EMT6 tumors was isolated by high salt extraction, the ECM used to coat chambered cover slip slides (μ-slides, Ibidi). Total interference reflection fluorescence (TIRF) microscopy was used to visualize collagen fibres (white arrow heads). βAPN treatment also reduced crosslinking in 4T1 and MT6 tumors (2).
Z-Projections 6 slides, z-distance 1μm, Scale bars: 10 μm. Error bars: ±SEM, n = 3.

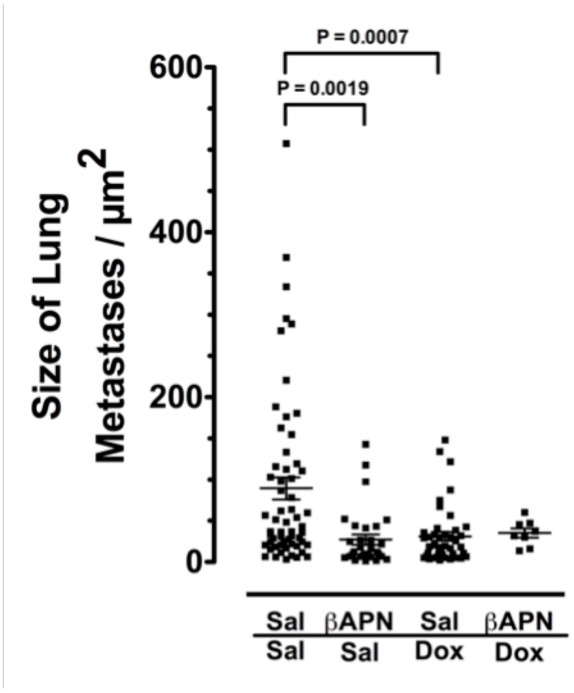

Supplemental Figure S3: Size distribution of 4T1 lung metastases after βAPN/DOX treatment. Both, βAPN and DOX reduced the size of metastases. Combination of both decreased both number and size of remaining metastases strongly. Numbers of metastases in the combination treatment group were too low for a meaningful statistical analysis of the size distribution.
Error bars: ±SEM.

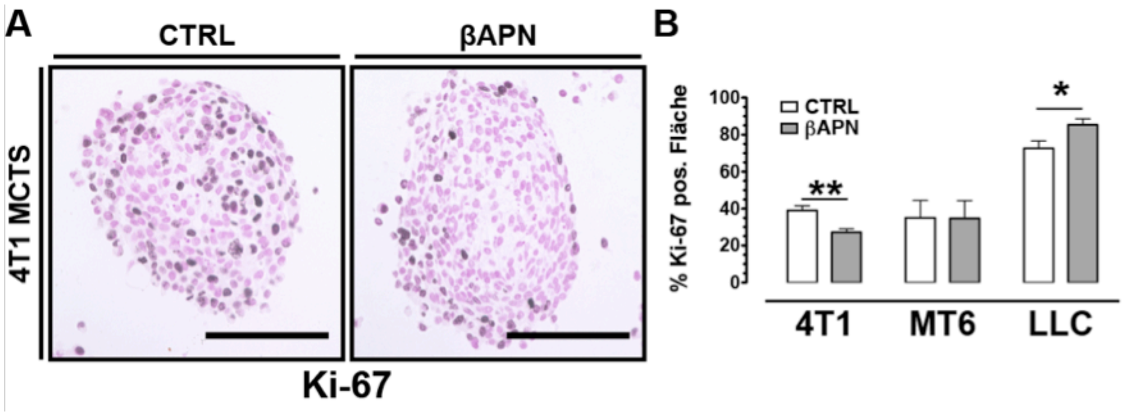

Supplemental Figure S5: Proliferation in MCTS after LOX(L)-inhibition with βAPN.

(A) Immunostaining of 4T1 MCTS for KI-67. SB = 100 µm.

(B) Quantification of Ki-67 positive cells in MCTS. Error bars: ±SEM. n = 4-10.

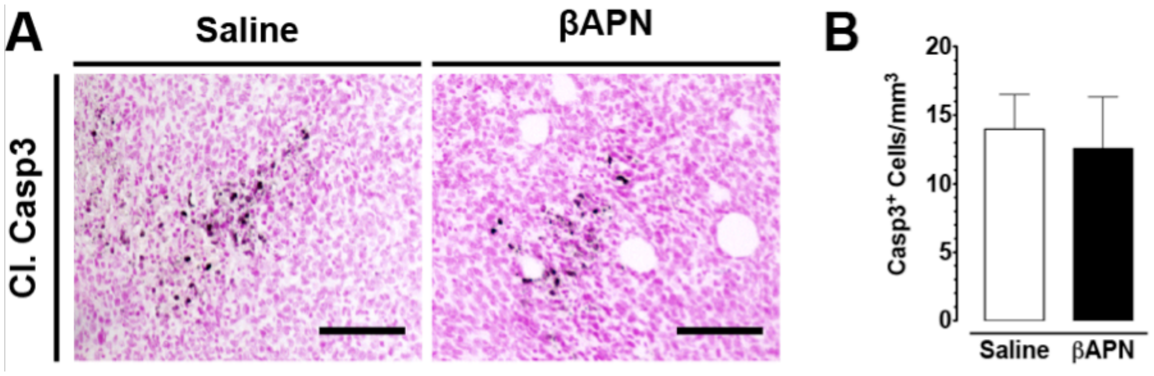

Supplemental Figure S4: Evaluation of apoptosis in 4T1 tumors after LOX(L)-inhibition with βAPN.

(A) Immunostaining of 4T1 tumor sections for the apoptosis marker cleaved caspase 3. SB = 100 µm.

(B) Quantification of cleaved caspase3 positive cells in 4T1 tumor sections. Error bars: ±SEM. n = 8.

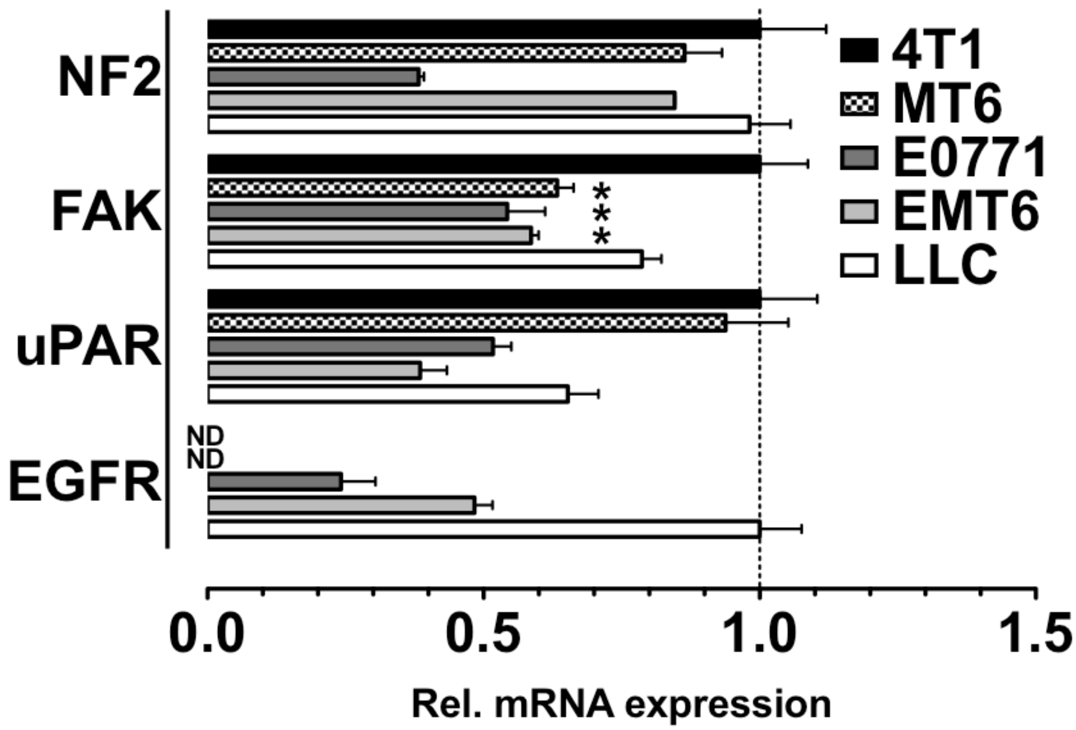

Supplemental Figure S6: Expression profile of FAK-sensitivity related genes in murine tumor cells.

mRNA levels of NF2/Merlin FAK, uPAR and EGFR was quantified using the GeXP multiplex analysis system. 4T1 cells, the sole line sensitive to FAK-inhibition with PF-573228, does not express lower levels of NF2, one known mediator of FAK-sensitivity (1). However, FAK-levels were significantly higher in 4T1 cells, than in PF-573228 resistant cells. N = 3, *: $p < 0.05$. ND: not detectable. Error bars: ±SEM. n = 8.

References

1. Shapiro IM, Kolev VN, Vidal CM, Kadariya Y, Ring JE, Wright Q, et al. Merlin deficiency predicts FAK inhibitor sensitivity: a synthetic lethal relationship. Sci Transl Med. 2014;6(237):237ra68.
2. Rohrig F, Vorlova S, Hoffmann H, Wartenberg M, Escorcia FE, Keller S, et al. VEGF-ablation therapy reduces drug delivery and therapeutic response in ECM-dense tumors. Oncogene. 2017;36(1):1-12.
3. Hatzis C, Pusztai L, Valero V, Booser DJ, Esserman L, Lluch A, et al. A genomic predictor of response and survival following taxane-anthracycline chemotherapy for invasive breast cancer. Jama. 2011;305(18):1873-81.
4. Itoh M, Iwamoto T, Matsuoka J, Nogami T, Motoki T, Shien T, et al. Estrogen receptor (ER) mRNA expression and molecular subtype distribution in ER-negative/progesterone receptor-positive breast cancers. Breast Cancer Res Treat. 2014;143(2):403-9.
5. Tsuji S, Midorikawa Y, Takahashi T, Yagi K, Takayama T, Yoshida K, et al. Potential responders to FOLFOX therapy for colorectal cancer by Random Forests analysis. British journal of cancer. 2012;106(1):126-32.
6. Yu KD, Zhu R, Zhan M, Rodriguez AA, Yang W, Wong S, et al. Identification of prognosis-relevant subgroups in patients with chemoresistant triple-negative breast cancer. Clin Cancer Res. 2013;19(10):2723-33.
7. Tabchy A, Valero V, Vidaurre T, Lluch A, Gomez H, Martin M, et al. Evaluation of a 30-gene paclitaxel, fluorouracil, doxorubicin, and cyclophosphamide chemotherapy response predictor in a multicenter randomized trial in breast cancer. Clin Cancer Res. 2010;16(21):5351-61.
8. Partheen K, Levan K, Osterberg L, Horvath G. Expression analysis of stage III serous ovarian adenocarcinoma distinguishes a sub-group of survivors. European journal of cancer. 2006;42(16):2846-54.
9. Wilmarth KR, Froines JR. In vitro and in vivo inhibition of lysyl oxidase by aminopropionitriles. J Toxicol Environ Health. 1992;37(3):411-23.
10. Zhang Y, Strehin I, Bedelbaeva K, Gourevitch D, Clark L, Leferovich J, et al. Drug-induced regeneration in adult mice. Sci Transl Med. 2015;7(290):290ra92.
11. Bianchi FT, Camera P, Ala U, Imperiale D, Migheli A, Boda E, et al. The collagen chaperone HSP47 is a new interactor of APP that affects the levels of extracellular beta-amyloid peptides. PLoS One. 2011;6(7):e22370.
12. Rani CS, Abe A, Chang Y, Rosenzweig N, Saltiel AR, Radin NS, et al. Cell cycle arrest induced by an inhibitor of glucosylceramide synthase. Correlation with cyclin-dependent kinases. J Biol Chem. 1995;270(6):2859-67.
